# Predicted Preferences for Tuberculosis Point-of-Care Tests Among Tuberculosis-Affected Individuals in 5 High Burden Countries

**DOI:** 10.1093/cid/ciag022

**Published:** 2026-01-14

**Authors:** Talemwa Nalugwa, Kinari M Shah, Danaida Marcelo, Robinah Nakawunde, Trang Trinh, Jerusha Emmanuel, Annet Nakaweesa, Ann Schraufnagel, Alfred Andama, Devasahayam J Christopher, Dinh Van Luong, Grant Theron, William Worodria, Maria del Mar Castro, Payam Nahid, Claudia M Denkinger, Adithya Cattamanchi, Charles Yu, Emily Kendall, Achilles Katamba, Andrew D Kerkhoff

**Affiliations:** Uganda Tuberculosis Implementation Research Consortium, Walimu, Kampala, Uganda; Center for Tuberculosis, Institute for Global Health Sciences, University of California SanFrancisco, San Francisco, California, USA; Division of HIV, Infectious Diseases and Global Medicine, SanFrancisco General Hospital, University of California San Francisco, San Francisco, California, USA; College of Medicine, De La Salle Medical and Health Sciences Institute, Cavite, Philippines; DSI-NRF Centre of Excellence for Biomedical Tuberculosis Research, South African Medical Research Council Centre for Tuberculosis Research, Division of Molecular Biology and Human Genetics, Faculty of Medicine and Health Sciences, Stellenbosch University, Cape Town, South Africa; UCSF - VNTP Research Collaboration Unit, Hanoi, Vietnam Center for Promotion of Advancement of Society, Hanoi, Vietnam; Department of Pulmonary Medicine, Christian Medical College, Vellore, India; Uganda Tuberculosis Implementation Research Consortium, Walimu, Kampala, Uganda; Division of Pulmonary and Critical Care Medicine, SanFrancisco General Hospital, University of California San Francisco, San Francisco, California, USA; Department of Medicine, Makerere University College of Health Sciences, Kampala, Uganda; Department of Pulmonary Medicine, Christian Medical College, Vellore, India; National Lung Hospital, Hanoi, Vietnam; DSI-NRF Centre of Excellence for Biomedical Tuberculosis Research, South African Medical Research Council Centre for Tuberculosis Research, Division of Molecular Biology and Human Genetics, Faculty of Medicine and Health Sciences, Stellenbosch University, Cape Town, South Africa; Division of HIV, Infectious Diseases and Global Medicine, SanFrancisco General Hospital, University of California San Francisco, San Francisco, California, USA; Department of Medicine, Makerere University College of Health Sciences, Kampala, Uganda; Department of Infectious Disease and Tropical Medicine, Medical Faculty University of Heidelberg, Heidelberg, Germany; German Center of Infection Research, Partner Site Heidelberg, Heidelberg, Germany; Center for Tuberculosis, Institute for Global Health Sciences, University of California SanFrancisco, San Francisco, California, USA; Institute for Global Health Sciences, University of California SanFrancisco, San Francisco, California, USA; Department of Infectious Disease and Tropical Medicine, Medical Faculty University of Heidelberg, Heidelberg, Germany; German Center of Infection Research, Partner Site Heidelberg, Heidelberg, Germany; Center for Tuberculosis, Institute for Global Health Sciences, University of California SanFrancisco, San Francisco, California, USA; Division of Pulmonary Diseases and Critical Care Medicine, University of California Irvine, Irvine, California, USA; De La Salle Medical and Health Sciences Institute, Cavite, Philippines; Division of Infectious Diseases, Department of Medicine, Johns Hopkins School of Medicine, Baltimore, Maryland, USA; Uganda Tuberculosis Implementation Research Consortium, Walimu, Kampala, Uganda; Clinical Epidemiology and Biostatistics Unit, Department of Medicine, Makerere University College of Health Sciences, Kampala, Uganda; Center for Tuberculosis, Institute for Global Health Sciences, University of California SanFrancisco, San Francisco, California, USA; Division of HIV, Infectious Diseases and Global Medicine, SanFrancisco General Hospital, University of California San Francisco, San Francisco, California, USA

**Keywords:** tuberculosis, diagnosis, point-of-care test, preference, discrete choice experiment

## Abstract

Point-of-care (POC) tests are needed to improve tuberculosis (TB) detection, but how TB-affected persons value POC tests is unknown. Simulations using discrete choice experiment-based data from 1149 TB-affected participants across 5 countries demonstrated that POC TB tests were highly preferred, even with modestly lower accuracy compared to standard facility-based testing.

Approximately 2.4 million people with active tuberculosis (TB) remain undiagnosed annually, representing the largest gap in the global TB care cascade [1]. Current TB diagnostic pathways in high-burden settings rely heavily on facility-based testing with Xpert MTB/RIF Ultra, requiring sputum collection and typically next-day results. While Xpert achieves high sensitivity, this approach may create barriers, including the inability to produce sputum, multiple clinic visits, and ultimately delayed diagnosis and treatment initiation.

Point-of-care (POC) TB diagnostics, such as urine-based TB-LAM tests, and emerging near-POC swab-based tests hold tremendous promise for addressing these barriers and closing the diagnostic gap by bringing rapid test results closer to TB-affected individuals [2], particularly in resource-limited settings. However, emerging POC technologies may have lower accuracy compared to centralized laboratory testing and may be perceived differently than standard-of-care (SOC) approaches, including lower trust for new technologies [3, 4]. We conducted simulations based on a previously published discrete choice experiment (DCE) across 5 high-burden countries to quantify the relative preferences of TB-affected individuals for POC TB tests compared to standard facility-based approaches. This secondary analysis also includes additional community-based participants from Uganda not previously reported.

## METHODS

Between December 2022 and September 2024, a survey-based DCE on TB diagnostic test features was administered to adults (≥18 years) with presumptive or confirmed TB at outpatient public health facilities across 5 high-burden countries (Philippines, Vietnam, South Africa, Uganda, and India) [5]. An additional 105 previously unreported participants were enrolled from a community-based TB case-finding study in Uganda to evaluate whether preferences differed by enrollment setting [6].

The DCE design has previously been described in detail [5]. In brief, the final design included 5 TB diagnostic test features with 3–4 levels each: (1) sample type (sputum, tongue swab, urine, and fingerprick blood), (2) accuracy (described as sensitivity; 70%, 80%, and 90%), (3) cost ($0, $2, and $4 USD), (4) location (facility, community site, and home), (5) time to result (15 minutes, 3 hours, and next day) (Supplementary File 1). Attributes and levels were selected through literature review, World Health Organization (WHO) target product profile (TPP) guidance [7, 8], and discussions with in-country research partners. Participants completed 12 random choice tasks featuring 2 different TB test options, where they were asked to (1) select their preferred test option and (2) indicate whether they would actually get tested using their preferred option. The DCE was near balanced and designed using Sawtooth Software 9.14.2.

Prior to conducting formal analysis, data quality measures were applied to improve data quality as previously described [9]. Hierarchical Bayes estimations were used to derive mean preference weights (MPWs) for each test feature level. Simulations using individual-level MPWs, including a “none” MPW derived from the follow-up intent question in each choice task, were conducted with the Sawtooth Choice Simulator tool to estimate relative preferences for different POC tests versus standard TB testing or neither option. The simulator used the shares of preference (logit rule) model to convert individual total utilities to probabilistic choice shares (totaling 100% for both test options) with 95% CIs.

## RESULTS

A total of 1149 participants were enrolled who met data quality standards. Overall, the median age was 42 (interquartile range [IQR], 29–54) years, 50.4% were female, 9.1% were Human Immunodeficiency Virus (HIV) positive, 13.1% had diabetes, and 32.7% had known current or prior TB. There were 207 participants from India, 210 from the Philippines, 219 from South Africa, 305 from Uganda (200 facility-based and 105 community-based), and 208 from Vietnam. Participant characteristics and MPWs by country are shown in Supplementary Tables 1 and 2, respectively.

If implemented at healthcare facilities, shares of preferences for POC tests compared to SOC testing were 78.8% (95% CI, 77.9–79.8), 58.1% (95% CI, 56.6–59.6), and 41.9% (95% CI, 40.0–43.8) if POC testing had the same, 10% less, or 20% lower sensitivity, respectively. Corresponding preferences were 70.0% (95% CI, 68.5–71.5), 50.7% (95% CI, 49.0–52.4), and 36.3% (95% CI, 34.4–38.2), respectively, if POC tests for TB were available in community settings, and 64.6% (95% CI, 62.7–66.5), 47.8% (95% CI, 45.9–49.7), and 34.4% (95% CI, 32.5–36.4), respectively, if available for home use. When compared to SOC testing, POC testing (15-minute results) consistently demonstrated higher preference shares than same-day testing (3-hour results), with preference increases of 8.6%–11.9% points across all accuracy levels and implementation settings (Supplementary Table 3). The proportion choosing neither test was <1.5% across all scenarios, and sample type had minimal impact on preferences, with tongue swab and sputum-based testing differing by 0%–1.6% points (Supplementary Table 3).

**Figure 1.**
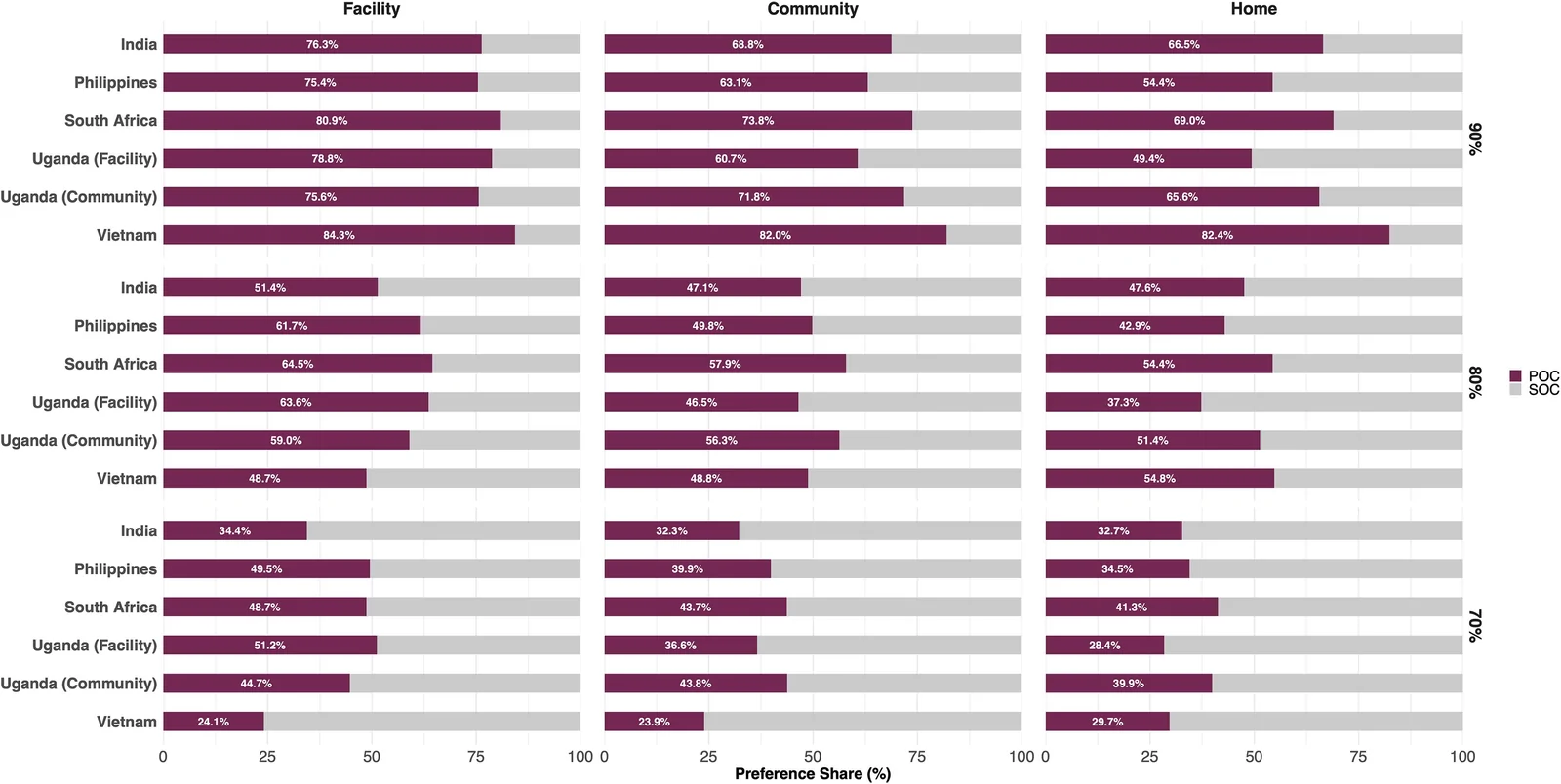
Predicted preferences for point-of-care (POC) TB tests implemented across different settings compared to a typical standard-of-care (SOC) testing strategy. SOC TB testing features were defined as sputum reliance, 90% accuracy, no cost, facility-based, and next-day results, mimicking centralized Xpert Ultra [10]. Point-of-care tests were assumed to provide results within 15 min and be available at no cost. Columns indicate hypothetical location for POC testing, and rows indicate hypothetical POC test sensitivity. Proportions indicate shares of preferences for each testing option, with the left portion of each bar (darker shading) indicating preference for POC and the right portion (lighter shading) indicating preference for SOC. Note that SOC also includes those who selected “neither,” as this represented <1.5% of participants across all scenarios.

Preferences for POC tests varied substantially by country (Figure 1), with Vietnam demonstrating the strongest preferences across all scenarios. Country-specific differences were most pronounced at lower accuracy levels, where at 70% sensitivity, preferences for POC testing ranged from 23.9% (Vietnam) to 43.7% (South Africa) for community-based testing and 28.2% (Uganda) to 41.2% (South Africa) for home-based testing (Supplementary Table 3). Community-enrolled participants in Uganda showed higher preferences for nearly all community- and home-based testing options compared to facility-enrolled participants from Uganda (range difference, 7.2%–17.8% points) (Figure 1; Supplementary Table 3).

## DISCUSSION

Our DCE-based simulations among persons accessing TB testing across 5 high-burden countries demonstrate that rapid POC diagnostics are highly valued by TB-affected adults. When POC tests approximated Xpert Ultra sensitivity (90%), they captured the majority of preference shares, whether made available at health facilities, at community sites, or at home. Even when sensitivity dropped by up to 20%, 24%–51% of participants across all countries still preferred POC TB testing across implementation settings. Furthermore, participants across all countries had a significantly greater preference for rapid (15-minute) compared to same-day (3-hour) results when simulated against the SOC. These data align with similarly strong preferences for same-day results identified in a previous DCE among people with TB in Zambia [10], underscoring that fast results are a dominant driver of preferences related to the TB diagnosis process. Our data further highlight that a meaningful proportion of people affected by TB would opt for decentralized, rapid tests despite moderate reductions in accuracy.

The sustained preference for POC testing, even at lower sensitivities approximating the lower bounds of current TPPs (65%–75%) [8], supports deployment of POC diagnostics at lower-level health facilities and as part of community case-finding campaigns and outreach events. While the only current commercially available POC test, Determine TB-LAM [11], falls short of these accuracy thresholds, emerging near-POC diagnostics such as MTB Ultima and MiniDock MTB show promising accuracy and alignment with the preferences of TB-affected persons for rapid testing [12]. Notably, TB-LAM's limited real-world use predominantly reflects its performance and associated narrow use rather than acceptability to people with TB. By bringing TB tests with fewer infrastructure requirements and rapid, on-the-spot results closer to where people live and work, programs can reduce current barriers related to diagnosis and pretreatment loss to follow-up [13, 14]. Moreover, our finding that sample type had minimal impact on choices suggests that nonsputum specimens (eg, tongue swabs) could be leveraged to improve patient comfort and increase the proportion able to provide a quality sample, with little effect on acceptability, especially when combined with efforts to raise trust and confidence in new technologies and alternative sample types [3, 4]. However, as experience with TB-LAM demonstrates, the impact of emerging POC and near-POC tests will greatly depend on implementation strategies that facilitate their uptake and routine use.

In Uganda, participants enrolled from community sites demonstrated notably stronger preferences for decentralized testing options compared to facility-enrolled participants in the same setting, which has several implications. First, this suggests that preferences for community- and home-based POC testing may be underestimated in our predominantly facility-based sample. This difference may reflect where individuals are in their disease course—those with mild or no symptoms identified through active case finding may place greater value on the convenience of community-based testing and early diagnosis [3, 4]. Ultimately, these findings highlight the importance of incorporating community perspectives when designing strategies to better reach and diagnose the 2+ million people who remain undiagnosed annually [1] and who likely have distinct preferences compared to people who present for care at health facilities.

Our study has several strengths, including enrollment of a large number of TB-affected individuals using standardized procedures across 5 high-burden countries with diverse healthcare contexts and rigorous data quality controls. Limitations include that we did not include drug susceptibility testing as an attribute in the DCE, so we were unable to simulate its impact on preferences. Given that current and emerging POC/near-POC tests lack this important feature, its impact on preferences warrants evaluation in future studies. Additionally, the extended enrollment period (2022–2024) may have introduced temporal variation in preferences, though we believe this is unlikely, as methods were consistent throughout and no major changes to local diagnostic practices occurred during this time. Finally, the use of stated rather than revealed preferences (inherent to all DCEs) and the use of simulated preference shares based on DCE-derived utilities may not fully align with actual uptake in the real world. However, DCEs have been shown to have good prediction accuracy for real-world choices in many health-related studies [15].

In high-burden settings, TB-affected individuals prioritize rapid, accessible diagnostics, often accepting modest sensitivity reductions. Implementation strategies that decentralize POC testing to align with these preferences offer a promising approach for closing current diagnostic gaps by improving detection among vulnerable populations facing barriers to care.

## Supplementary Material

ciag022_Supplementary_Data

## Supplementary Data

Supplementary materials are available at *Clinical Infectious Diseases* online. Consisting of data provided by the authors to benefit the reader, the posted materials are not copyedited and are the sole responsibility of the authors, so questions or comments should be addressed to the corresponding author.

## References

[1] World Health Organization . *Global Tuberculosis report 2025*. World Health Organization; 2025. Available at: https://www.who.int/teams/global-programme-on-tuberculosis-and-lung-health/tb-reports/global-tuberculosis-report-2025

[2] Vachaspathi T. *Tuberculosis diagnostics pipeline report 2025.* Treatment Action Group; 2025. Available at: https://www.treatmentactiongroup.org/resources/pipeline-report/2025-pipeline-report/. (Accessed December 10, 2025).

[3] Sales A, Le H, Muzazu S, et al “That is why I trust”: a qualitative study on acceptability and feasibility of novel tongue swab diagnostics to assess people presenting with tuberculosis symptoms in Viet Nam and Zambia. medRxiv [Preprint]. Available from: doi:10.1101/2025.06.30.25330535. Published online January 1, 2025 PMC13536577 42321775

[4] Castro MDM, Le H, Manoj Kumar K, et al Informing people-centered target product profiles for TB diagnostics: a multi-country qualitative study. medRxiv [Preprint]. [cited 2025 Jul 14]. Available from: doi:10.1101/2025.07.11.25331385. Published online January 1, 2025

[5] Shah K . Preferences for diagnostic test features among people testing for tuberculosis: a multi-country discrete choice experiment. Lancet Prim Care In Press.

[6] Sung J, Nantale M, Nalutaaya A, et al Evidence for tuberculosis in individuals with Xpert Ultra “trace” sputum during screening of high-burden communities. Clin Infect Dis Off Publ Infect Dis Soc Am 2024; 78:723–9. 10.1093/cid/ciad595 PMC10954329 37787077

[7] World Health Organization . *High priority target product profiles for new tuberculosis diagnostics: report of a consensus meeting*. World Health Organization; 2014. https://www.who.int/publications/i/item/WHO-HTM-TB-2014.18

[8] World Health Organization . *Target product profiles for tuberculosis diagnosis and detection of drug resistance*; 2024.

[9] Shah K, Nalugwa T, Marcelo D, et al Preferences for tuberculosis diagnostic test features among people tested for tuberculosis: a multicountry discrete choice experiment. Lancet Prim Care 2025; 1:100012.

[10] Kerkhoff AD, Kagujje M, Nyangu S, et al Pathways to care and preferences for improving tuberculosis services among tuberculosis patients in Zambia: a discrete choice experiment. PLoS One 2021; 16:0252095. 10.1371/journal.pone.0252095 PMC8407587 34464392

[11] World Health Organization . Consolidated guidelines on tuberculosis: rapid diagnostics for tuberculosis detection. Geneva, Switzerland: World Health Organization, 2024. https://www.who.int/publications/i/item/9789240089488

[12] Steadman A, Kumar KM, Asege L, et al Diagnostic accuracy of swab-based molecular tests for tuberculosis using near-point-of-care platforms: a multi-country evaluation. EBioMedicine 2025; 121:105991. 41175672 10.1016/j.ebiom.2025.105991 PMC12617638

[13] Khosa C, Cossa M, Leukes V, et al Implementing the Molbio Truenat platform and tuberculosis assays versus standard of care at primary care clinics for the detection and treatment of tuberculosis in Mozambique and Tanzania (TB-CAPT CORE): a cluster-randomised trial. Lancet Prim Care 2025; 1:100028.

[14] Lessells RJ, Cooke GS, McGrath N, Nicol MP, Newell ML, Godfrey-Faussett P. Impact of point-of-care Xpert MTB/RIF on tuberculosis treatment initiation. A cluster-randomized trial. Am J Respir Crit Care Med 2017; 196:901–10. 28727491 10.1164/rccm.201702-0278OC PMC5649979

[15] Zhang Y, Anh Ho TQ, Terris-Prestholt F, et al Prediction accuracy of discrete choice experiments in health-related research: a systematic review and meta-analysis. eClinicalMedicine 2025; 79:102965. 39791109 10.1016/j.eclinm.2024.102965 PMC11714376

