# Supplementary material for: Predicted Preferences for Tuberculosis Point-of-Care Tests Among Tuberculosis-Affected Individuals in 5 High Burden Countries
Source: Clin Infect Dis. 2026 Jan 14;83(2):e395–8. doi: 10.1093/cid/ciag022 (PMC13537115; doi:10.1093/cid/ciag022)
Supplement: Supplementary file 1 [file ciag022_supplementary_data.zip › Supplemental Tables.docx]

| **Table S1. Participant characteristics by country** | | | | | | | |  |
| --- | --- | --- | --- | --- | --- | --- | --- | --- |
|  | **Total** | **India** | **Philippines** | **South Africa** | **Uganda** | | **Vietnam** | |
|  |  |  |  |  | **Facility** | **Community** |  |  |
| **N (%)** | **1,149**  **(100.0%)** | **207**  **(19.8%)** | **210**  **(20.1%)** | **219**  **(20.8%)** | **200**  **(19.4%)** | **105**  **(9.1%)** | **208**  **(20.1%)** | |
| **Age, median (IQR)** | 42 (29-54) | 48 (35-60) | 43 (31-57) | 36 (27-45) | 34 (27-42) | 33 (25-40) | 54 (39-64) | |
| **Age category** |  |  |  |  |  |  |  | |
| <25 | 161 (14.0) | 18 (8.7) | 29 (13.8) | 41 (18.7) | 34 (17.0) | 25 (23.8) | 14 (6.7) | |
| 25-34 | 265 (23.1) | 32 (15.5) | 37 (17.6) | 59 (27.0) | 70 (35.0) | 38 (36.2) | 29 (13.9) | |
| 35-44 | 249 (21.7) | 39 (18.8) | 43 (20.5) | 63 (28.8) | 56 (28.0) | 25 (23.8) | 23 (11.1) | |
| 45-54 | 203 (17.7) | 46 (22.2) | 39 (18.6) | 34 (15.5) | 31 (15.5) | 13 (12.4) | 40 (19.2) | |
| 55-64 | 155 (13.5) | 41 (19.8) | 32 (15.2) | 17 (7.7) | 7 (3.5) | 3 (2.9) | 55 (26.4) | |
| 65+ | 116 (10.1) | 31 (14.9) | 30 (14.3) | 5 (2.3) | 2 (1.0) | 1 (1.0) | 47 (22.6) | |
| **Sex** |  |  |  |  |  |  |  | |
| Male | 570 (49.6) | 113 (54.6) | 96 (45.7) | 85 (38.8) | 88 (44.0) | 63 (60.0) | 125 (60.1) | |
| Female | 579 (50.4) | 94 (45.4) | 114 (54.3) | 134 (61.2) | 112 (56.0) | 42 (40.0) | 83 (39.9) | |
| **Relationship status** |  |  |  |  |  |  |  | |
| Single | 347 (30.2) | 28 (13.5) | 61 (29.1) | 153 (69.9) | 54 (27.0) | 23 (21.9) | 28 (13.5) | |
| Currently married | 524 (45.6) | 164 (79.2) | 84 (40.0) | 32 (14.6) | 29 (14.5) | 38 (36.2) | 177 (85.1) | |
| Widowed | 57 (5.0) | 15 (7.3) | 19 (9.1) | 8 (3.6) | 13 (6.5) | 1 (1.0) | 1 (0.5) | |
| Divorced or  separated | 67 (5.8) | 0 | 6 (2.9) | 6 (2.7) | 41 (20.5) | 12 (11.4) | 2 (1.0) | |
| Stable relationship but not married | 154 (13.4) | 0 | 40 (19.1) | 20 (9.1) | 63 (31.5) | 31 (29.5) | 0 | |
| **Education level** |  |  |  |  |  |  |  | |
| Never attended school | 42 (3.7) | 25 (12.1) | 1 (0.5) | 2 (1.0) | 10 (5.0) | 3 (2.9) | 1 (0.5) | |
| Some primary school | 91 (7.9) | 11 (5.3) | 8 (3.8) | 5 (2.3) | 48 (24.0) | 16 (15.2) | 3 (1.4) | |
| Primary school | 163 (14.2) | 28 (13.5) | 24 (11.4) | 35 (16.0) | 39 (19.5) | 21 (20.0) | 16 (7.7) | |
| Some secondary school | 255 (22.2) | 25 (12.1) | 111 (52.9) | 22 (10.1) | 47 (23.5) | 21 (20.0) | 29 (13.9) | |
| Secondary school | 359 (31.2) | 44 (21.3) | 22 (10.5) | 139 (63.5) | 32 (16.0) | 32 (30.5) | 90 (43.3) | |
| Post-secondary education (university or college) | 239 (20.8) | 74 (35.8) | 44 (20.9) | 16 (7.3) | 24 (12.0) | 12 (11.4) | 69 (33.2) | |
| **TB History^** |  |  |  |  |  |  |  | |
| No TB | 773 (67.3) | 141 (68.1) | 112 (53.3) | 145 (66.2) | 117 (58.5) | 102 (97.1) | 156 (75.0) | |
| Current TB | 38 (3.3) | 10 (4.8) | 11 (5.2) | 6 (2.7) | 11 (5.5) | 0 | 0 (0.0) | |
| Prior TB | 338 (29.4) | 56 (27.1) | 87 (41.4) | 68 (31.1) | 72 (36.0) | 3 (2.9) | 52 (25.0) | |
| **HIV status*** |  |  |  |  |  |  |  | |
| No | 878 (76.4) | 150 (72.5) | 193 (91.9) | 164 (74.8) | 153 (76.5) | 99 (94.3) | 117 (56.3) | |
| Yes | 104 (9.1) | 0 (0.0) | 3 (1.4) | 55 (25.1) | 46 (23.0) | 0 | 2 (1.0) | |
| Unknown | 167 (14.5) | 57 (27.5) | 14 (6.7) | 0 (0.0) | 1 (0.5) | 6 (5.7) | 89 (42.8) | |
| **Diabetes history*** |  |  |  |  |  |  |  | |
| No | 977 (85.0) | 154 (74.4) | 155 (73.8) | 206 (94.1) | 173 (86.5) | 105 (0.0) | 184 (88.5) | |
| Yes | 150 (13.1) | 49 (23.7) | 42 (20.0) | 13 (5.9) | 26 (13.0) | 0 | 20 (9.6) | |
| Unknown | 22 (1.9) | 4 (1.9) | 13 (6.2) | 0 | 1 (0.5) | 0 | 4 (1.9) | |
| **Distance from facility** (n=858, missing 291) |  |  |  |  |  |  |  | |
| <5 km | 332 (38.7) | 10 (5.0) | 23 (23.9) | 177 (90.3) | 56 (50.5) | 26 (50.0) | 40 (19.6) | |
| 5-10km | 181 (21.1) | 12 (6.0) | 65 (67.7) | 14 (7.1) | 32 (28.8) | 17 (32.7) | 41 (20.1) | |
| 10-20km | 105 (12.2) | 32 (16.1) | 6 (6.3) | 3 (1.5) | 21 (18.9) | 7 (13.5) | 36 (17.6) | |
| 20-50km | 103 (12.0) | 34 (17.1) | 1 (1.0) | 1 (0.5) | 1 (0.9) | 2 (3.8) | 64 (31.4) | |
| >50km | 137 (16.0) | 111 (55.8) | 1 (1.0) | 1 (0.5) | 1 (0.9) | 0 | 23 (11.3) | |

Medians (IQR) are presented for continuous variables that were not normally distributed. We did not collect data on race and ethnicity as such classifications were not consistently defined across countries. ^TB status reflects self-reported history and available clinical records at the time of DCE completion. Some participants may have had undiagnosed TB at the time of the DCE and were diagnosed subsequently. However, because our interest was in how confirmed TB diagnosis or treatment history influences test preferences, these individuals were classified as having “no known history of TB. 53 participants with no history of TB had undiagnosed, prevalent TB at the time of the DCE but it was diagnosed subsequently. However, because our interest was in how confirmed TB diagnosis or treatment history influences test preferences, these individuals were classified as having “no known history of TB”.

**Table S2. The mean preference weights for TB test features and their options among included participants (n=1,149)**

|  | **Overall**  **(n=1,149)** | **India**  **(n=207)** | **Philippines**  **(n=210)** | **South Africa**  **(n=219)** | **Uganda** **(n=305)** | | **Vietnam**  **(n=208)** |
| --- | --- | --- | --- | --- | --- | --- | --- |
|  |  |  |  |  | **Facility**  **(n=200)** | **Community**  **(n=105)** |  |
| **Mean preference weights for TB test feature options** | | | | | | | |
| **Sample type** | | | | | | | |
| **Sputum** | 4.0  (1.9, 6.1) | 0.8  (-4.7, 6.3) | 1.6  (-3.3, 6.6) | 6.3  (1.6,10.9) | 6.6  (2.2,11.0) | 1.1  (-7.6, 9.8) | 6.0  (2.2,9.9) |
| **Tongue swab** | 2.8  (1.5, 4.1) | 2.3  (-0.8, 5.3) | 4.7  (1.8, 7.5) | 1.5  (-0.6, 3.6) | 4.2  (1.9, 6.4) | -5.2  (-13.5, 3.0) | 5.5  (3.5, 7.6) |
| **Urine** | -4.1  (-5.8, -2.5) | -2.4  (-6.7, 1.8) | -2.8  (-7.1, 1.5) | -5.1  (-8.7, -1.5) | -3.1  (-6.2, -0.1) | -7.6  (-15.1, -0.1) | -5.2  (-8.4, -2.0) |
| **Fingerprick** | -2.7  (-4.8, -0.6) | -0.6  (-6.3, 5.0) | -3.5  (-8.3, 1.2) | -2.7  (-7.4, 2.0) | -7.6  (-11.7, -3.6) | 11.7  (2.2, 21.3) | -6.4  (-10.4, -2.3) |
| **Accuracy** | | | | | | | |
| **70%** | -56.5  (-59.5, -53.6) | -65.5  (-72.6, -58.5) | 38.4  (-44.6, -32.2) | -46.7  (-53.1, -40.3) | -42.9  (-50.0, -35.7) | -44.3  (-52.1, -36.5) | -95.6  (-100.9, -90.2) |
| **80%** | 0.9  (0.2, 1.6) | 1.7  (-0.2, 3.6) | 0.8  (-1.2, 2.7) | 1.2  (-0.3, -2.7) | 1.5  (0.0, 3.0) | 1.4  (-1.0, 3.7) | -1.0  (-2.3, 0.3) |
| **90%** | 55.7  (52.6, 58.7) | 63.8  (56.7, 71.0) | 37.6  (31.2, 44.1) | 45.5  (38.9, 52.1) | 41.3  (34.1, 48.6) | 42.9  (34.8, 51.0) | 96.6  (91.2, 102.0) |
| **Location** | | | | | | | |
| **Home** | -16.3  (-19.2, -13.5) | -7.4  (-13.7, -1.1) | -30.5  (-36.9, -24.2) | -12.0  (-17.7, -6.3) | -47.9  (-56.1, -39.6) | -9.9  (-14.8, -5.0) | 11.6  (6.4,16.7) |
| **Community location** | -3.2  (-4.5, -1.9) | -5.9  (-9.3, -2.5) | -4.5  (-7.9, -1.2) | -3.4  (-5.9, -0.9) | -0.7  (-3.3, 1.9) | 11.2  (6.9, 15.5) | -8.6  (-11.0, -6.1) |
| **Health facility** | 19.5  (16.7, 22.4) | 13.3  (6.4, 20.1) | 35.1  (29.2, 41.0) | 15.4  (9.7, 21.1) | 48.6  (40.4, 56.8) | -1.3  (-7.8, 5.2) | -3.0  (-8.0, -2.0) |
| **Cost** | | | | | | | |
| **Free** | 62.3  (59.3, 65.4) | 49.9  (42.9, 56.8) | 74.9  (68.0, 81.8) | 75.9  (68.5, 83.3) | 67.3  (60.3, 74.2) | 85.3  (73.6, 97.0) | 31.4  (26.6, 36.3) |
| **$2** | -2.4  (-3.3, -1.4) | -4.2  (-6.6, -1.8) | -4.7  (-7.0, -2.4) | -0.6  (-2.3, 1.1) | -2.5  (-4.3, -0.6) | 0.1  (-4.7, 5.0) | -1.2  (-3.0, 0.5) |
| **$4** | -60.0  (-63.0, -57.0) | -45.7  (-52.5, -38.9) | -70.2  (-77.3, -63.2) | -75.3  (-82.6, -68.0) | -64.8  (-71.6, -57.9) | -85.4  (-95.8, -75.0) | -30.2  (-34.8, -25.6) |
| **Time** | | | | | | | |
| **15 minutes** | 38.2  (36.3, 40.0) | 34.2  (29.8, 38.5) | 34.9  (30.3, 39.5) | 43.4  (39.5, 47.3) | 38.0  (34.0, 42.1) | 20.5  (14.9, 26.1) | 49.0  (45.0, 53.1) |
| **3 hours** | 3.8  (3.0, 4.7) | 3.9  (1.9, 6.0) | 2.2  (-0.3, 4.6) | 1.9  (0.1, 3.8) | 3.7  (2.1, 5.3) | 11.5  (8.3, 14.7) | 3.6  (1.8, 5.4) |
| **Next day** | -42.0  (-43.8, -40.2) | -38.1  (-42.5, -33.7) | -37.1  (-41.4, -32.7) | -45.4  (-49.4, -41.3) | -41.7  (-45.8, -37.7) | -31.9  (-37.2, -26.6) | -52.6  (-56.7, -48.6) |

**Table S3.** **Predicted preference for point-of-care tuberculosis test compared to standard-of-care tuberculosis test by country.** Values indicate shares of preference. For each simulation, a hypothetical TB test was compared against a TB test representative of the current standard of care (Xpert) with the following levels: sputum-based, facility-based, 90% accurate, next day results, and free. The hypothetical comparator TB test assumed testing was free.

|  | **Overall**  **(n=1,149)** | **India**  **(n=207)** | **Philippines**  **(n=210)** | **South Africa**  **(n=219)** | **Uganda**  **(n=305)** | | **Vietnam**  **(n=208)** |
| --- | --- | --- | --- | --- | --- | --- | --- |
|  |  |  |  |  | **Facility (n=200)** | **Community**  **(n=105)** |  |
| **Facility** | | | | | | | |
| Sp+90%+SD | 70.2  (69.3-71.0) | 68.5  (66.5-70.5) | 67.0  (65.0-69.0) | 71.2  (69.3-73.0) | 70.7  (68.9-72.5) | 69.2  (66.6-71.8) | 74.0  (72.0-75.9) |
| Sp+90%+POC | 78.8  (77.9-79.8) | 76.3  (74.0-78.6) | 75.4  (72.9-77.8) | 80.9  (78.9-82.9) | 78.8  (76.8-80.9) | 75.6  (72.2-78.9) | 84.3  (82.2-86.4) |
| TS+90%+SD | 68.6  (67.5-69.7) | 67.3  (64.8-69.7) | 66.2  (63.3-69.0) | 68.6  (66.2-71.0) | 68.6  (66.2-71.0) | 66.8  (63.2-70.4) | 73.2  (70.8-75.6) |
| TS+90%+POC | 77.3  (76.2-78.5) | 75.5  (72.9-78.1) | 73.9  (70.8-77.0) | 78.7  (76.2-81.2) | 77.1  (74.7-79.5) | 73.7  (69.6-77.7) | 83.2  (80.7-85.8) |
| Sp+80%+SD | 46.2  (44.7-47.6) | 41.8  (38.5-45.1) | 50.9  (47.8-54.1) | 50.7  (47.6-53.9) | 53.1  (49.8-56.4) | 50.0  (45.5-54.4) | 32.4  (29.2-35.5) |
| Sp+80%+POC | 58.1  (56.6-59.6) | 51.4  (47.8-55.1) | 61.7  (58.2-65.2) | 64.5  (61.3-67.6) | 63.6  (60.1-67.1) | 59.0  (53.9-64.0) | 48.7  (44.9-52.5) |
| TS+80%+SD | 45.9  (44.4-47.5) | 42.1  (38.5-45.8) | 51.6  (48.0-55.3) | 49.3  (46.0-52.6) | 52.1  (48.5-55.7) | 48.4  (43.4-53.5) | 33.2  (29.7-36.7) |
| TS+80%+POC | 57.4  (55.8-59.0) | 51.5  (47.7-55.3) | 61.5  (57.6-65.4) | 62.7  (59.3-66.1) | 62.1  (58.3-65.9) | 57.5  (52.1-62.9) | 49.0  (45.0-53.0) |
| Sp+70%+SD | 32.5  (30.8-34.2) | 27.3  (23.7-31.0) | 39.7  (35.8-43.6) | 37.7  (33.8-41.7) | 41.5  (37.4-45.6) | 36.3  (31.0-41.6) | 14.4  (11.6-17.2) |
| Sp+70%+POC | 41.9  (40.0-43.8) | 34.4  (30.1-38.7) | 49.5  (45.3-53.8) | 48.7  (44.4-53.0) | 51.2  (46.8-55.6) | 44.7  (38.7-50.6) | 24.1  (20.2-28.0) |
| TS+70%+SD | 32.7  (31.0-34.4) | 27.9  (24.1-31.8) | 40.8  (36.7-45.0) | 36.8  (32.8-40.7) | 40.9  (36.8-45.0) | 35.8  (30.4-41.3) | 15.5  (12.4-18.6) |
| TS+70%+POC | 41.9  (39.9-43.8) | 34.6  (30.3-39.0) | 50.5  (46.0-55.0) | 47.7  (43.4-52.0) | 50.4  (45.9-54.9) | 44.0  (38.0-50.1) | 24.9  (20.9-29.0) |
| **Community** | | | | | | | |
| Sp+90%+ND | 42.1  (40.9-43.3) | 44.8  (41.6-47.9) | 36.5  (33.9-39.1) | 43.4  (40.8-46.0) | 33.5  (30.4-36.6) | 48.2  (44.3-52.0) | 49.0  (46.3-51.6) |
| Sp+90%+SD | 60.6  (59.1-62.1) | 61.2  (57.7-64.7) | 52.7  (49.4-56.0) | 63.0  (59.9-66.1) | 51.6  (47.6-55.6) | 65.4  (61.2-69.7) | 71.6  (68.7-74.4) |
| Sp+90%+POC | 70.0  (68.5-71.5) | 68.8  (65.2-72.4) | 63.1  (59.6-66.7) | 73.8  (70.7-76.9) | 60.7  (56.7-64.8) | 71.8  (67.3-76.3) | 82.0  (79.2-84.7) |
| TS+90%+ND | 41.8  (40.5-43.2) | 44.5  (41.3-47.7) | 37.2  (34.4-40.1) | 42.0  (39.2-44.8) | 33.0  (29.7-36.3) | 47.6  (43.2-51.9) | 49.3  (46.4-52.2) |
| TS+90%+SD | 60.0  (58.5-61.5) | 61.1  (57.6-64.5) | 53.1  (49.6-56.6) | 61.7  (58.5-64.9) | 50.3  (46.2-54.4) | 64.5  (59.9-69.2) | 71.3  (68.3-74.2) |
| TS+90%+POC | 69.5  (67.9-71.0) | 69.4  (65.9-72.8) | 63.0  (59.2-66.7) | 72.8  (69.6-76.0) | 59.3  (55.1-63.4) | 71.2  (66.3-76.1) | 81.5  (78.6-84.4) |
| Sp+80%+ND | 24.4  (23.1-25.6) | 25.1  (21.9-28.3) | 26.9  (23.9-29.9) | 28.5  (25.4-31.5) | 21.7  (18.8-24.6) | 32.2  (27.4-37.1) | 15.4  (13.3-17.5) |
| Sp+80%+SD | 39.4  (37.8-41.0) | 38.5  (34.5-42.5) | 39.2  (35.5-42.9) | 44.8  (41.2-48.5) | 36.8  (33.0-40.7) | 48.2  (42.8-53.6) | 32.9  (29.6-36.2) |
| Sp+80%+POC | 50.7  (49.0-52.4) | 47.1  (42.8-51.3) | 49.8  (45.8-53.9) | 57.9  (54.1-61.7) | 46.5  (42.2-50.7) | 56.3  (50.6-62.1) | 48.8  (44.8-52.9) |
| TS+80%+ND | 24.6  (23.3-25.9) | 25.3  (21.9-28.7) | 27.7  (24.6-30.8) | 27.8  (24.7-30.8) | 21.7  (18.8-24.7) | 32.7  (27.7-37.7) | 16.1  (13.8-18.5) |
| TS+80%+SD | 39.3  (37.7-41.0) | 38.1  (34.2-42.1) | 40.2  (36.3-44.1) | 44.0  (40.4-47.6) | 36.4  (32.4-40.4) | 47.5  (41.8-53.2) | 33.4  (29.9-36.9) |
| TS+80%+POC | 50.4  (48.7-52.2) | 46.6  (42.4-50.9) | 50.6  (46.3-54.8) | 57.0  (53.2-60.8) | 45.8  (41.4-50.1) | 55.9  (50.0-61.7) | 49.0  (44.8-53.1) |
| Sp+70%+ND | 16.9  (15.6-18.1) | 16.4  (13.4-19.4) | 21.3  (18.2-24.4) | 21.2  (18.1-24.3) | 16.0  (13.3-18.8) | 23.9  (18.8-29.0) | 5.6  (4.1-7.0) |
| Sp+70%+SD | 27.4  (25.7-29.0) | 25.8  (21.9-29.7) | 31.3  (27.3-35.3) | 33.4  (29.4-37.4) | 27.9  (24.2-31.7) | 36.0  (30.1-41.8) | 13.8  (11.1-16.5) |
| Sp+70%+POC | 36.1  (34.3-38.0) | 32.3  (27.8-36.8) | 39.9  (35.6-44.3) | 43.7  (39.3-48.0) | 36.6  (32.3-41.0) | 43.8  (37.5-50.1) | 23.9  (20.1-27.7) |
| TS+70%+ND | 17.2  (15.9-18.5) | 17.3  (14.0-20.6) | 21.9  (18.6-25.2) | 20.7  (17.6-23.8) | 16.0  (13.2-18.7) | 24.3  (19.2-29.4) | 6.4  (4.5-8.2) |
| TS+70%+SD | 27.5  (25.8-29.1) | 25.8  (21.9-29.8) | 32.2  (28.1-36.2) | 32.5  (28.7-36.4) | 27.8  (24.0-31.6) | 36.0  (30.2-41.8) | 14.6  (11.7-17.5) |
| TS+70%+POC | 36.3  (34.4-38.2) | 32.1  (27.6-36.6) | 41.3  (36.8-45.8) | 43.2  (38.8-47.5) | 36.3  (31.9-40.7) | 43.7  (37.3-50.1) | 24.5  (20.6-28.5) |
| **Home** | | | | | | | |
| Sp+90%+ND | 40.8  (39.2-42.4) | 45.2  (41.3-49.0) | 31.0  (27.7-34.2) | 41.4  (38.0-44.8) | 28.2  (24.4-32.0) | 44.0  (39.2-48.7) | 56.2  (52.5-59.9) |
| Sp+90%+SD | 55.8  (54.0-57.6) | 58.8  (54.7-62.9) | 44.2  (40.3-48.2) | 58.3  (54.4-62.2) | 41.9  (37.1-46.7) | 58.9  (53.6-64.1) | 73.7  (70.3-77.1) |
| Sp + 90% + POC | 64.6  (62.8-66.4) | 66.5  (62.5-70.6) | 54.4  (50.2-58.6) | 69.0  (65.2-72.9) | 49.4  (44.4-54.5) | 65.6  (60.2-71.1) | 82.4  (79.3-85.6) |
| TS+90%+ND | 40.7  (39.1-42.4) | 45.1  (41.2-49.0) | 32.2  (28.7-35.7) | 40.6  (37.0-44.1) | 27.7  (23.7-31.6) | 44.2  (39.4-49.1) | 55.9  (52.1-59.7) |
| TS+90%+SD | 55.6  (53.8-57.4) | 58.8  (54.7-62.9) | 45.2  (41.1-49.3) | 57.1  (53.2-61.1) | 40.9  (36.1-45.7) | 59.2  (53.8-64.6) | 73.5  (70.2-76.9) |
| TS+90%+POC | 64.6  (62.7-66.5) | 67.1  (63.0-71.2) | 54.7  (50.3-59.1) | 68.0  (64.1-72.0) | 48.7  (43.6-53.8) | 66.5  (60.8-72.2) | 82.8  (79.7-85.9) |
| Sp+80%+ND | 24.0  (22.6-25.5) | 26.7  (23.1-30.3) | 22.5  (19.4-25.6) | 27.6  (24.1-31.0) | 17.5  (14.4-20.6) | 29.9  (24.8-35.0) | 22.6  (19.5-25.6) |
| Sp+80%+SD | 37.6  (35.8-39.3) | 38.8  (34.7-42.9) | 33.2  (29.3-37.2) | 42.3  (38.3-46.3) | 29.6  (25.4-33.8) | 43.7  (37.9-49.5) | 40.3  (36.4-44.2) |
| Sp + 80% + POC | 47.9  (46.0-49.8) | 47.6  (43.1-52.0) | 42.9  (38.6-47.2) | 54.4  (50.3-58.5) | 37.3  (32.6-42.0) | 51.4  (45.1-57.6) | 54.8  (50.5-59.1) |
| TS+80%+ND | 24.4  (22.9-25.9) | 27.1  (23.3-30.9) | 23.6  (20.2-26.9) | 27.3  (23.8-30.8) | 17.6  (14.3-20.8) | 30.3  (25.2-35.4) | 23.1  (19.9-26.3) |
| TS+80%+SD | 37.6  (35.8-39.4) | 38.7  (34.4-42.9) | 34.5  (30.4-38.5) | 41.8  (37.8-45.9) | 29.1  (24.9-33.4) | 44.0  (38.1-50.0) | 40.2  (36.2-44.2) |
| TS+80%+POC | 47.8  (45.9-49.7) | 47.4  (42.9-52.0) | 43.6  (39.2-48.1) | 53.7  (49.4-57.9) | 36.5  (31.7-41.3) | 51.9  (45.6-58.2) | 55.1  (50.7-59.5) |
| Sp+70%+ND | 16.0  (14.7-17.3) | 17.4  (14.2-20.6) | 18.2  (15.1-21.4) | 20.3  (17.1-23.6) | 12.2  (9.5-14.9) | 21.9  (16.9-27.0) | 8.4  (6.4-10.4) |
| Sp+70%+SD | 25.6  (23.9-27.2) | 25.8  (21.9-29.7) | 26.8  (22.8-30.8) | 31.5  (27.4-35.5) | 21.4  (17.7-25.1) | 32.4  (26.6-38.2) | 18.4  (15.2-21.6) |
| Sp + 70% + POC | 34.0  (32.2-35.9) | 32.7  (28.2-37.3) | 34.5  (30.1-38.9) | 41.3  (36.8-45.8) | 28.4  (24.0-32.8) | 39.9  (33.5-46.4) | 29.7  (25.5-33.9) |
| TS+70%+ND | 16.0  (14.7-17.3) | 17.4  (14.2-20.6) | 18.2  (15.1-21.4) | 20.3  (17.1-23.6) | 12.2  (9.5-14.9) | 21.9  (16.9-27.0) | 8.4  (6.4-10.4) |
| TS+70%+SD | 25.6  (23.9-27.2) | 25.8  (21.9-29.7) | 26.8  (22.8-30.8) | 31.5  (27.4-35.5) | 21.4  (17.7-25.1) | 32.4  (26.6-38.2) | 18.4  (15.2-21.6) |
| TS+70%+POC | 34.4  (32.5-36.4) | 33.2  (28.5-37.9) | 35.7  (31.1-40.2) | 41.2  (36.7-45.7) | 28.2  (23.7-32.7) | 40.5  (33.9-47.0) | 30.2  (25.9-34.5) |

*Sp=sputum; TS=Tongue swab; SD=same-day (3 hours), POC=rapid results (15 mins). Note: Shares of preference sum to 100, comprising preferences for the POC test (shown), the standard-of-care test, and neither test. “Neither” was <1.5% across country-specific simulations.
