# Supplementary material for: Predicted Preferences for Tuberculosis Point-of-Care Tests Among Tuberculosis-Affected Individuals in 5 High Burden Countries
Source: Clin Infect Dis. 2026 Jan 14;83(2):e395–8. doi: 10.1093/cid/ciag022 (PMC13537115; doi:10.1093/cid/ciag022)
Supplement: Supplementary file 1 [file ciag022_supplementary_data.zip › Supplementary File 1.pdf]

R2D2 DCE Patient Education Booklet

We would like to ask you some questions to understand what you prefer when being tested for tuberculosis. It is a priority to improve tuberculosis tests for those undergoing testing. We want to make sure that future tuberculosis tests have qualities that individuals like yourself would prefer. Your answers to this survey may help scientists design tests that meet your preferences

UCSF

This survey will take approximately 30-45 minutes to complete. You may pause at any time if you become tired. However, all responses must be completed on the same day. Whether or not you choose to participate in this study will not impact your care at this facility.

Would you like to learn more?

We would like to make sure you that you understand that the survey and the options that we ask you give your opinion on are hypothetical, which means they will not affect your current TB treatment plan or overall healthcare in any way.

UCSF

- The first option for a new tuberculosis test is the sample type that you provide and that is tested for tuberculosis.
- We will ask you about four types of samples that you would prefer to provide for tuberculosis testing

Sample types

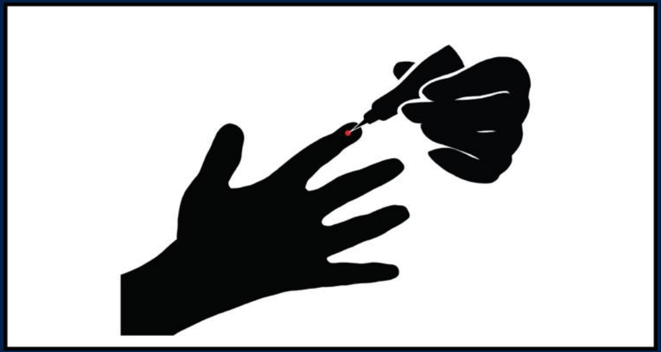

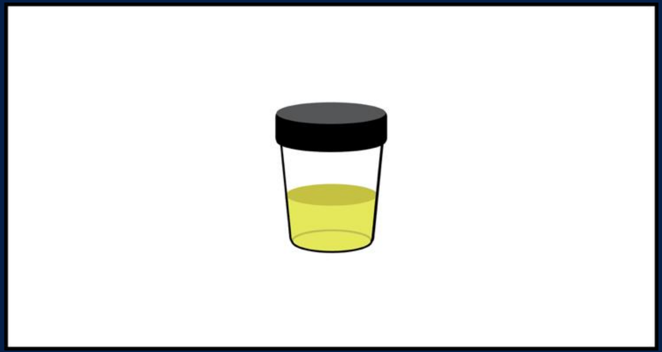

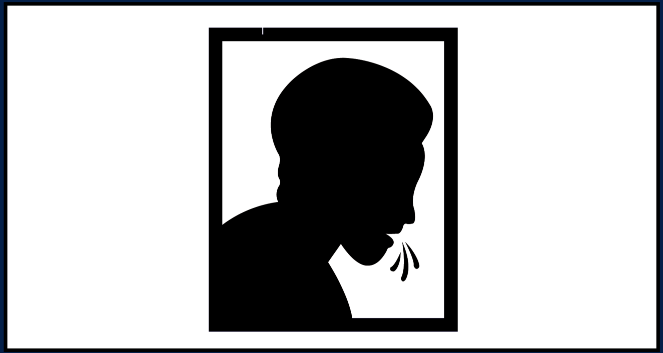

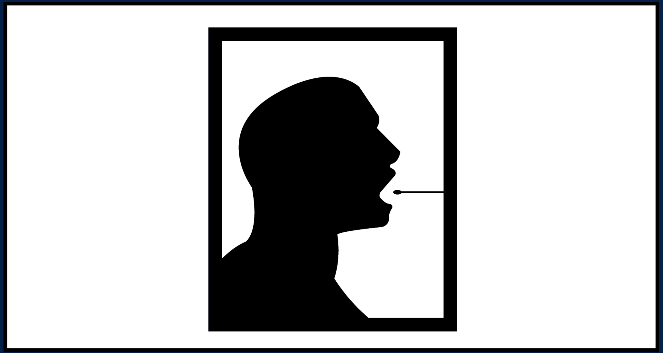

Sample type 1- Sputum

- The first sample type is sputum. This requires you to take deep breaths and cough hard until you feel a thick mucous in the back of your throat. This sample is then spit into a cup. This option looks like the graphic on the front of this card.

Sample type- Sputum

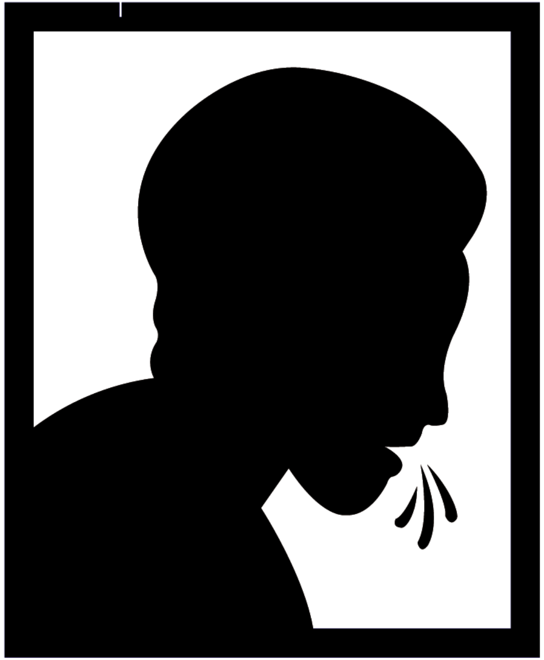

Sample type - Finger prick

- The second sample type is a blood finger prick. This requires using a very small, sharp blade to obtain a very small amount of blood from your finger.
- This option looks like the graphic on the front of this card.

Sample types- Finger prick

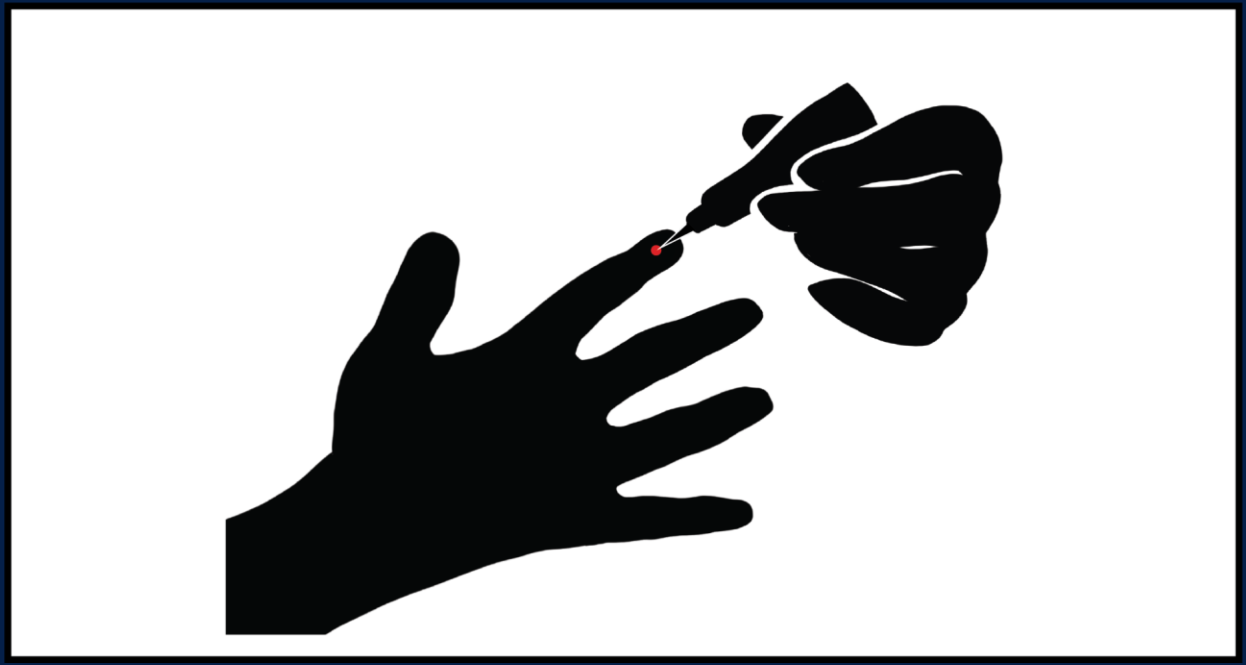

Sample type - Urine

- The third sample type is urine. This requires you to start peeing and then collect a small amount of pee in a plastic cup
- This option looks like the graphic on the front of this card.

Sample type- Urine

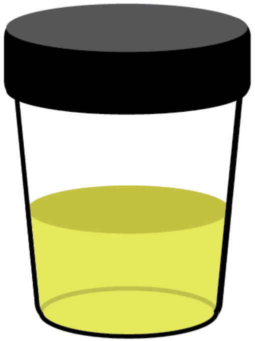

Sample type - Oral swab

- The fourth sample type is a tongue swab. This requires you to stick out your tongue so that it can be swabbed for about 15 seconds. This option looks like the graphic on the front of this card.

Sample types-Oral swab

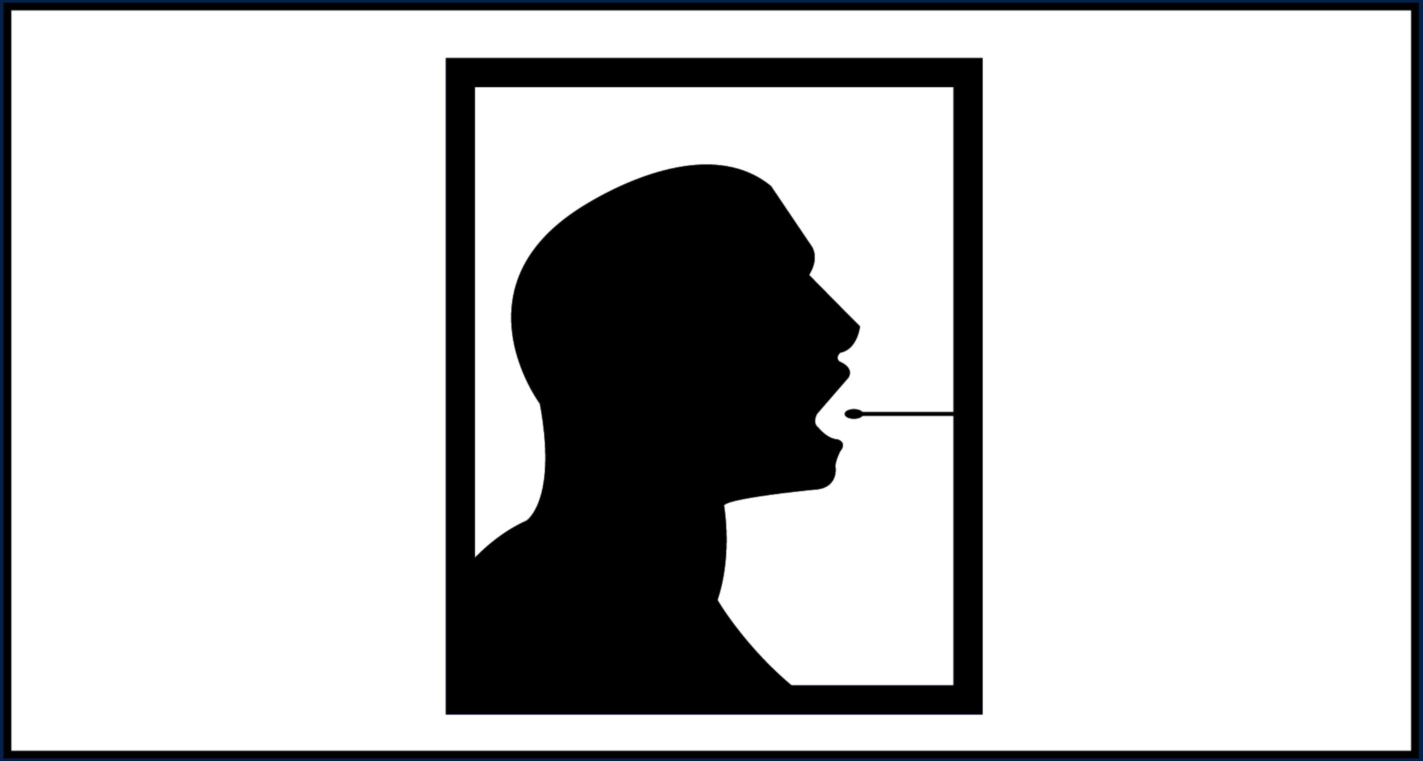

Sample types: Check understanding

- Do you have any questions about what these pictures mean?

Sample types

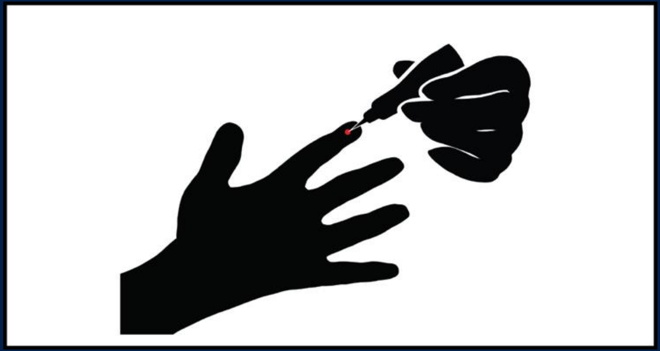

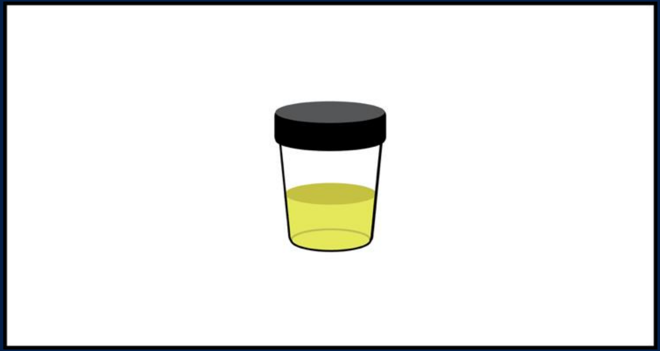

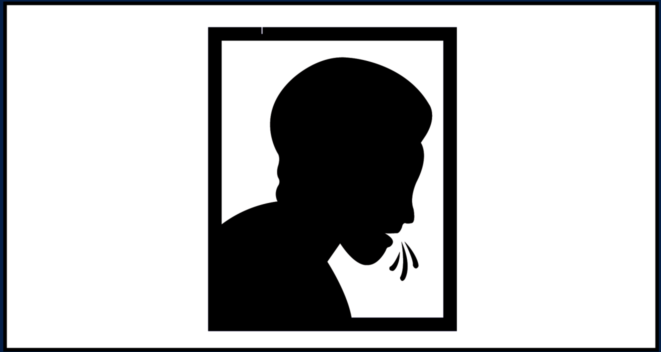

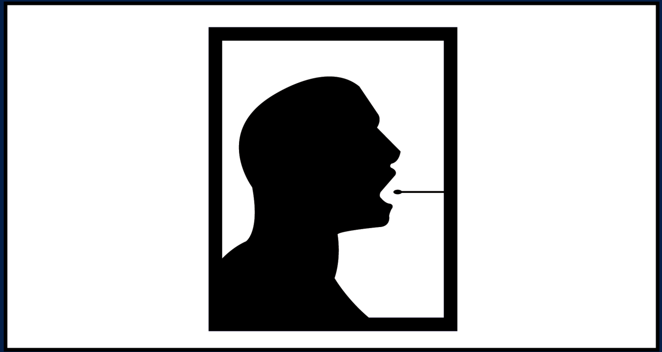

- The second option for a new tuberculosis test is how well the performs at detecting tuberculosis, also known as accuracy. We will ask you about different levels of accuracy that you would prefer for a tuberculosis test.

Accuracy

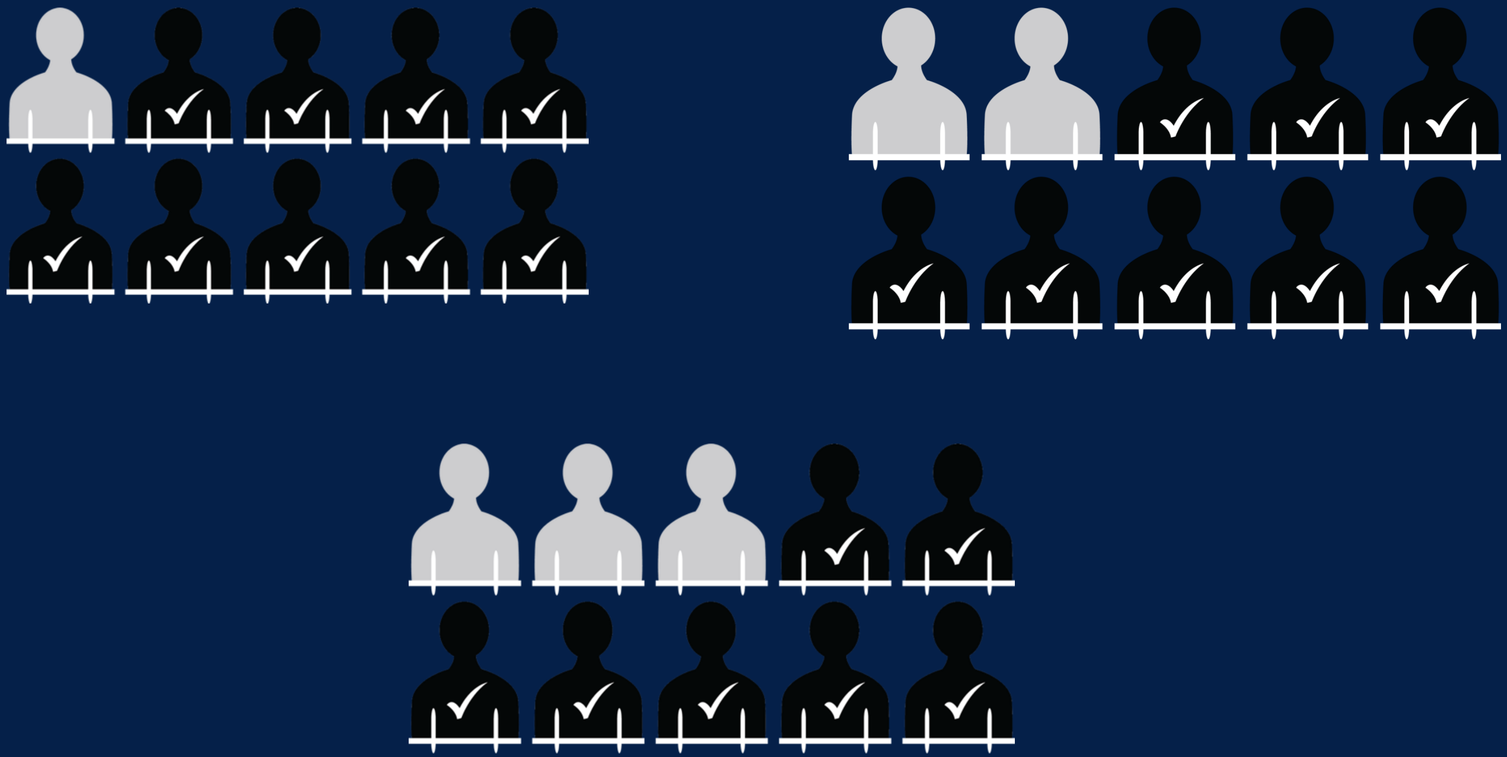

- The chance that the test misses tuberculosis when you have tuberculosis is: 1 in 10. This means that if 10 people had tuberculosis disease, the test would correctly detect tuberculosis in 9 persons, but would miss tuberculosis in 1 person. This option looks like the graphic on the front of this card.

Accuracy- 9 in 10

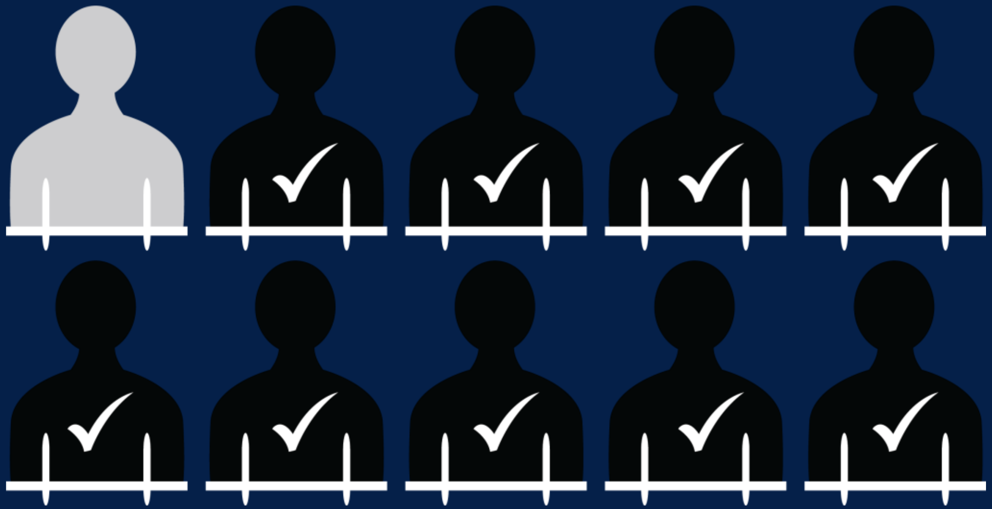

- The chance that the test misses tuberculosis when you have tuberculosis is: 2 in 10. This means that if 10 people had tuberculosis disease, the test would correctly detect tuberculosis in 8 persons, but would miss tuberculosis in 2 persons.
- This option looks like the graphic on the front of this card.

Accuracy- 8 in 10

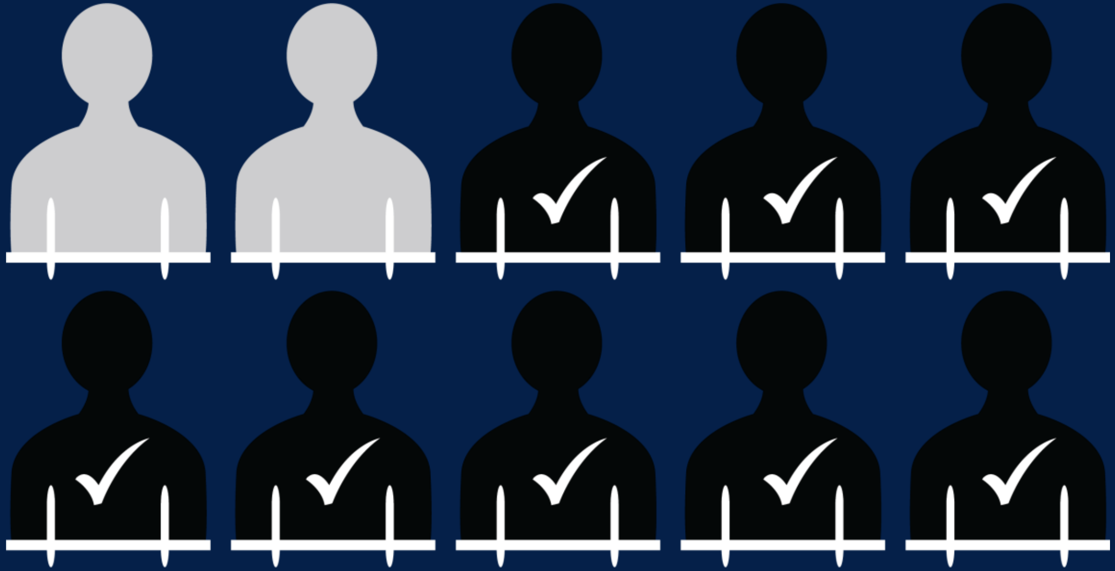

- The chance that the test misses tuberculosis when you have tuberculosis is: 3 in 10. This means that if 10 people had tuberculosis disease, the test would correctly detect tuberculosis in 7 persons, but would miss tuberculosis in 3 persons.
- This option looks like the graphic on the front of this card.

Accuracy- 7 in 10

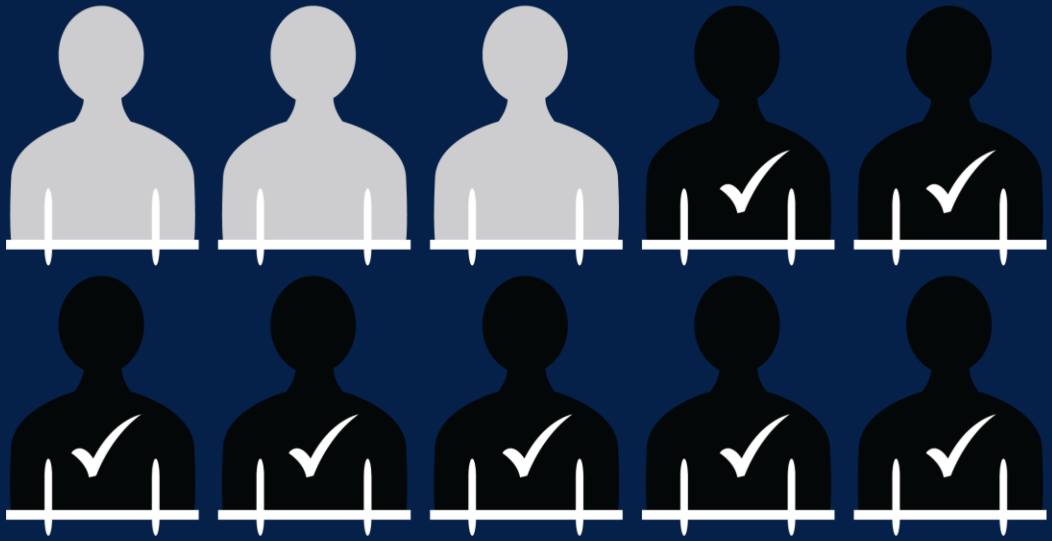

Accuracy: Check understanding

- Do you have any questions about what these pictures mean?

Accuracy

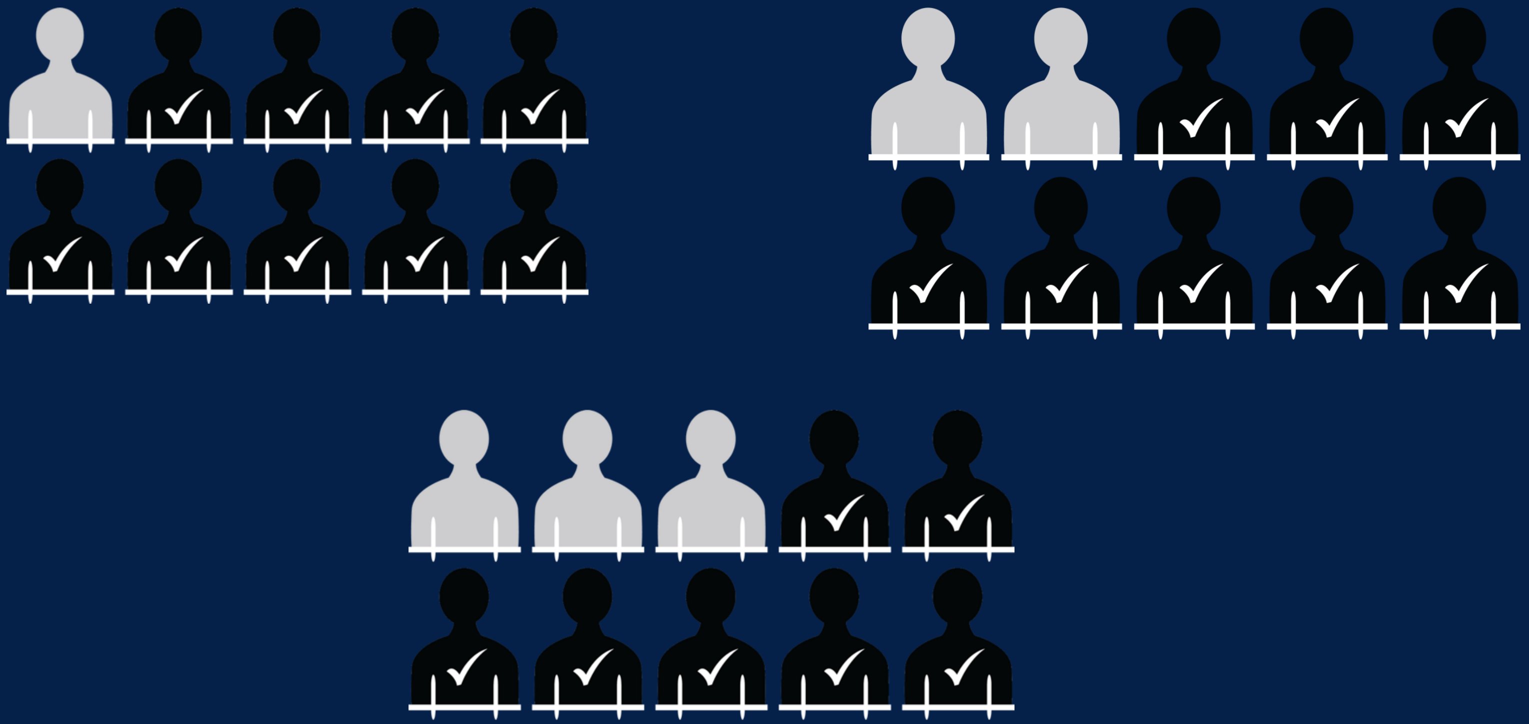

UCSF

Check understanding using 7 out of 10 graphic.

- What does this option mean?
  - Participant should say some variation of: this test would find 7 out of 10 TB cases, this test would miss 3 of 10 TB positive cases.

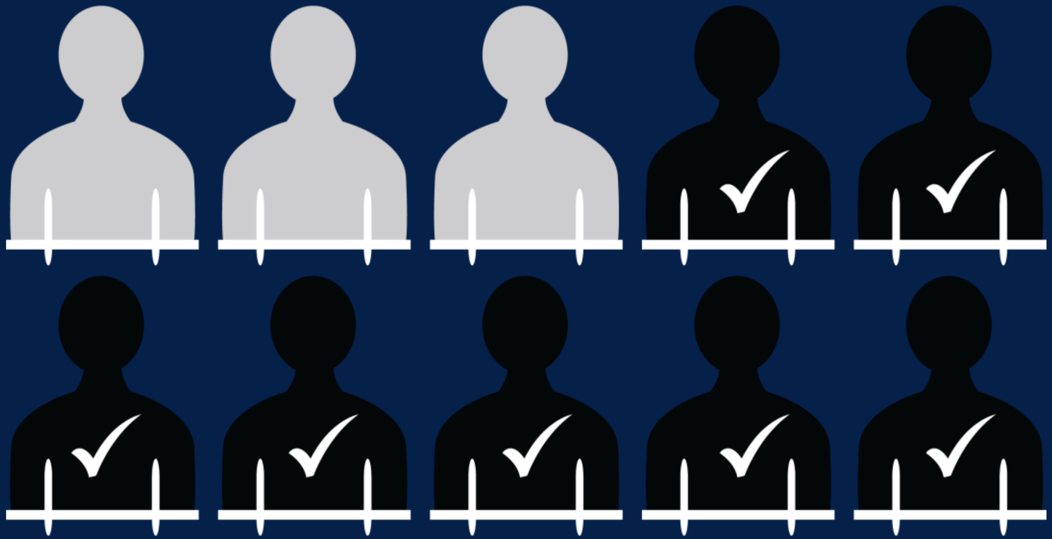

- The third option for a new tuberculosis test is the location where the tuberculosis test is performed. We will ask you about three different locations where you would prefer to undertake tuberculosis testing.

Location of testing

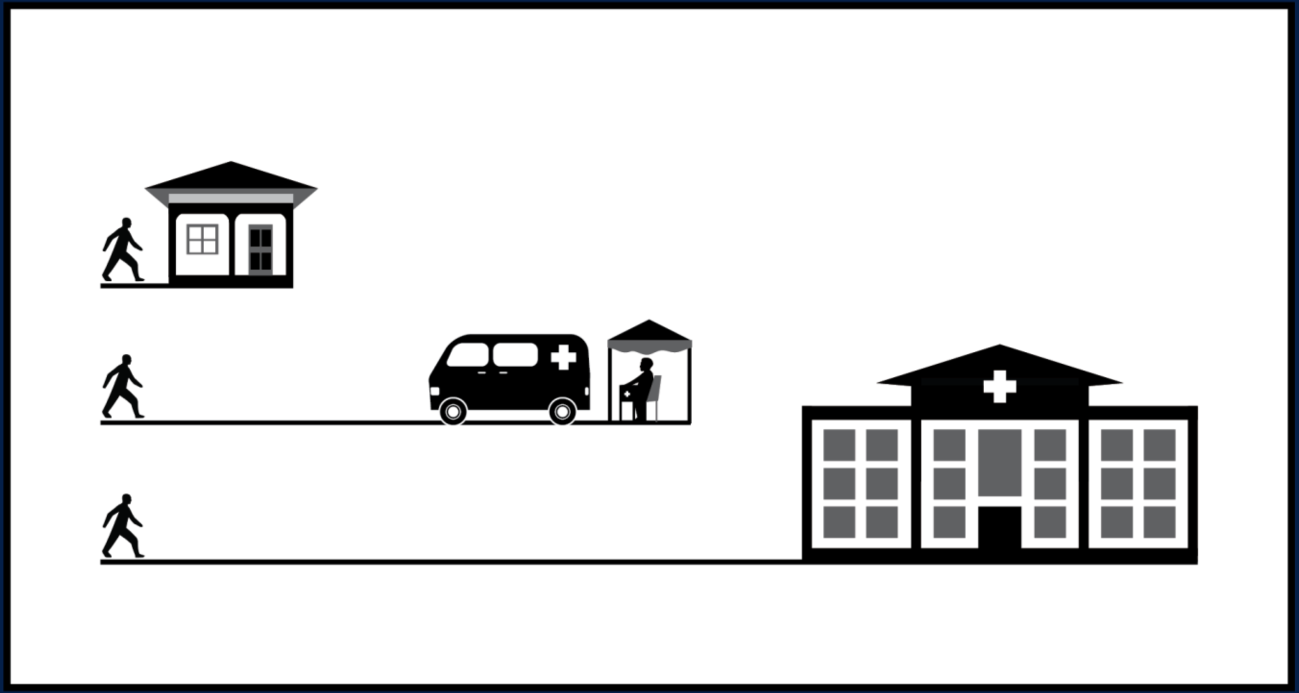

Home test

- The first location is your home. This means that a tuberculosis test could performed in your home. You must first get the test from elsewhere, but it can be performed at home at any time after you get the test.
- You can perform the test by yourself, or a trusted friend or family member can help you.

Location option 1- Home test

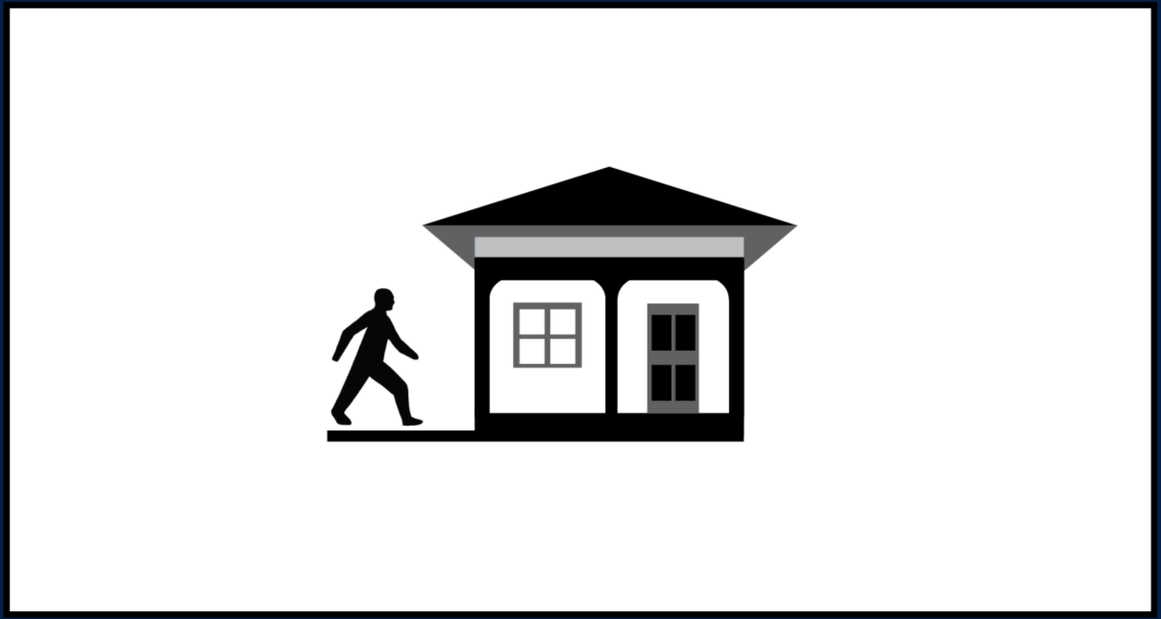

Community site

- The second location is a community site. This means that a tuberculosis test can be performed at a convenient location in your community, such as a smaller health clinic or pharmacy, or another community location that conducts health testing. This community location would not necessarily be affiliated with the nearest hospital or larger health facility.
- You must travel to the community site to get tested.

Location option 2- Community site

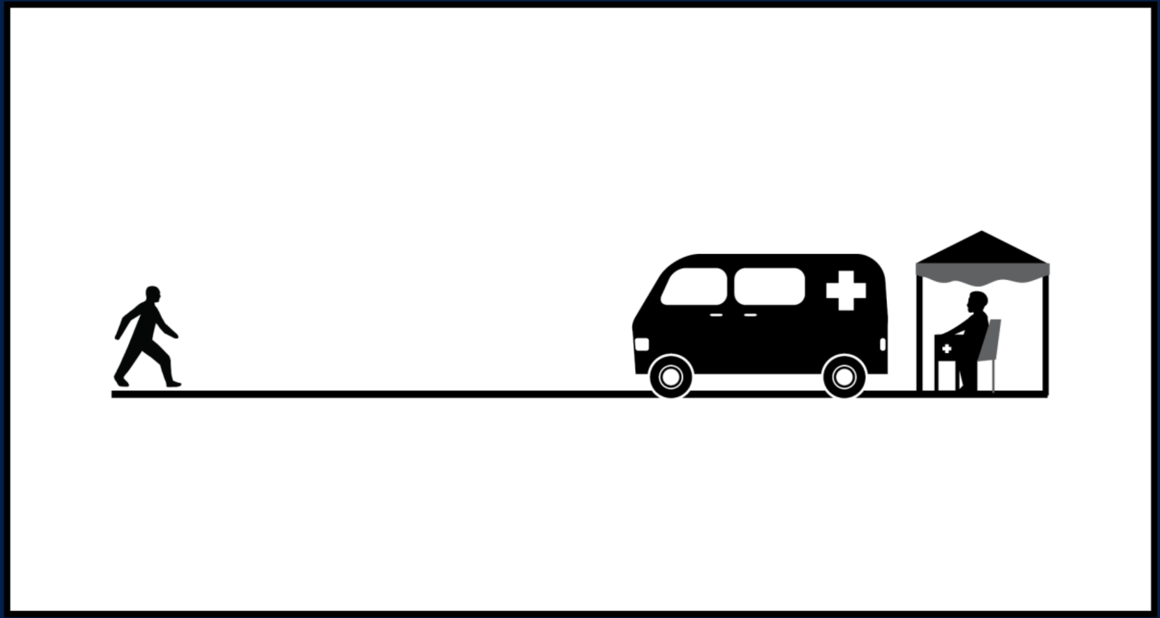

UCSF

Health facility or hospital

The third location is a health facility. This means that a tuberculosis test can only be performed at a health facility or a hospital, like this one. You must travel to the health facility to get tested.

Location option 3- Large health facility or Hospital

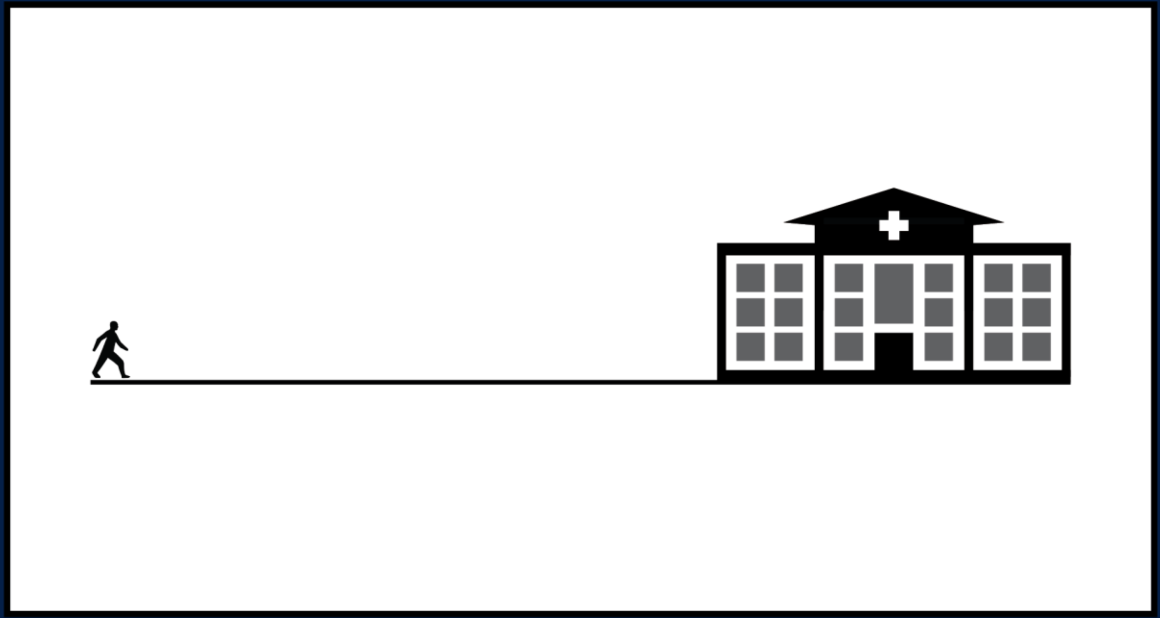

Check understanding of locations

- Do you have any questions about these pictures and what they represent?

Location of testing

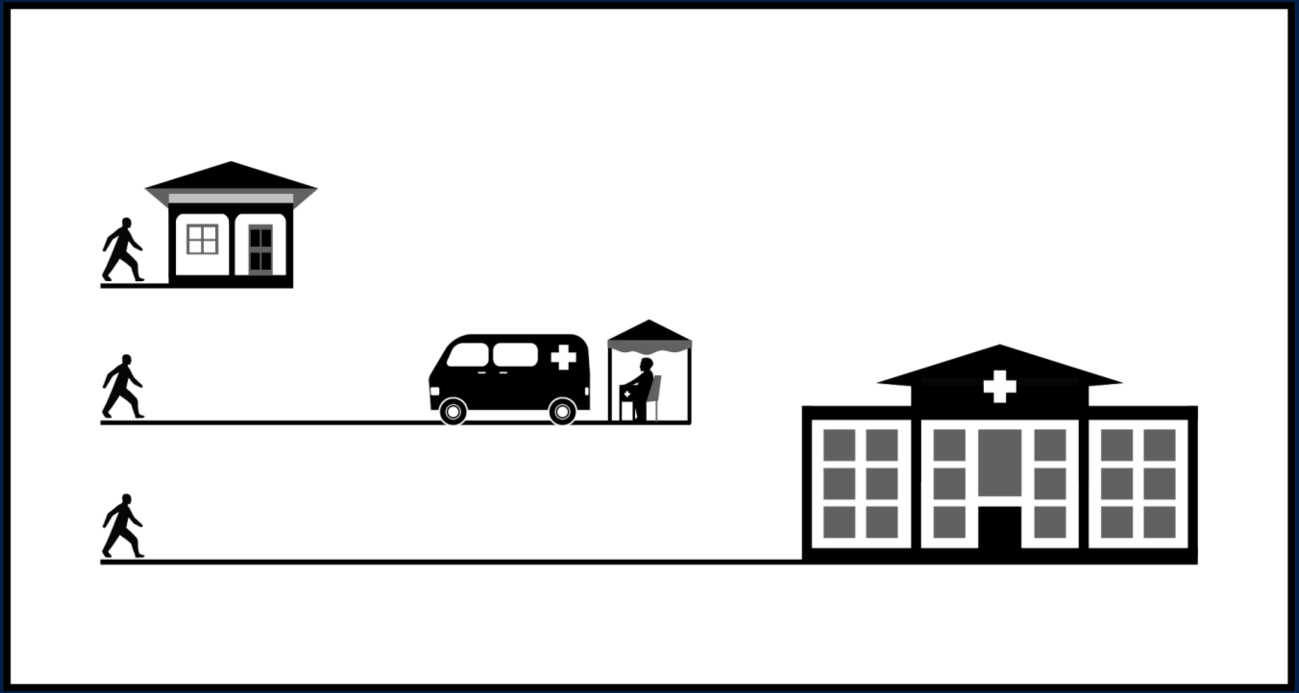

Time to results

- The fourth option for a new tuberculosis test is the time to results of your test. This is the amount of time it takes to receive the results of your tuberculosis test after you have provided a sample. We will ask you about three different durations that you would prefer for how long you need to wait for your test results.

Time to test result

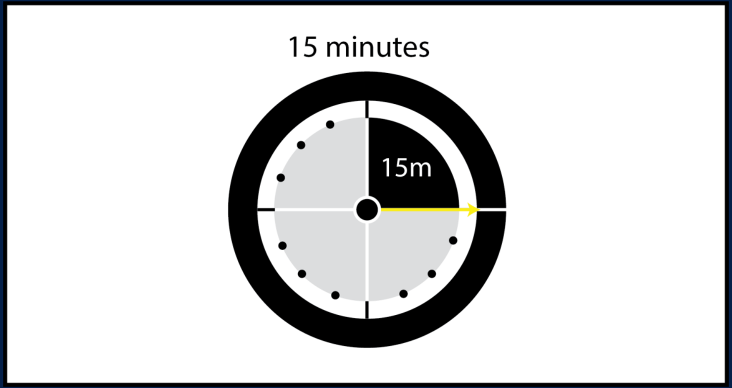

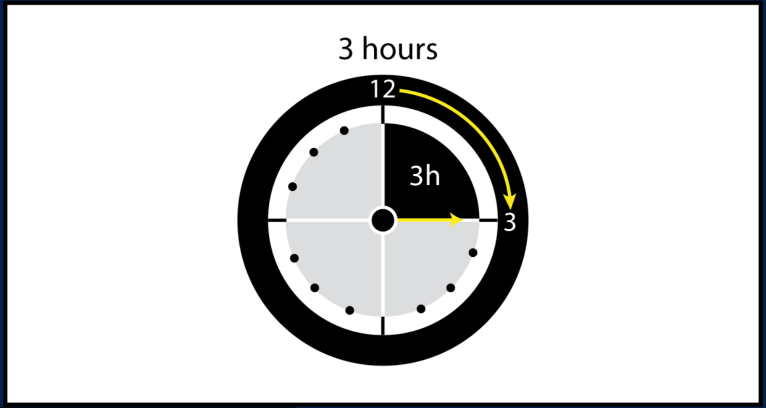

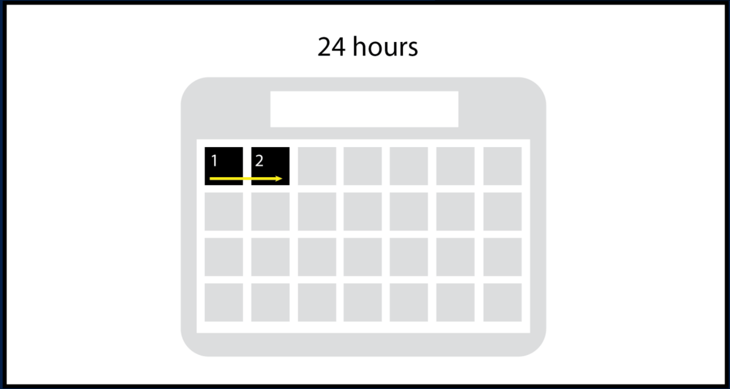

Time to results

- The first amount of time is 15 minutes. This means that after you provide a sample, the results of your tuberculosis test will be available in 15 minutes.

Time to test result-1

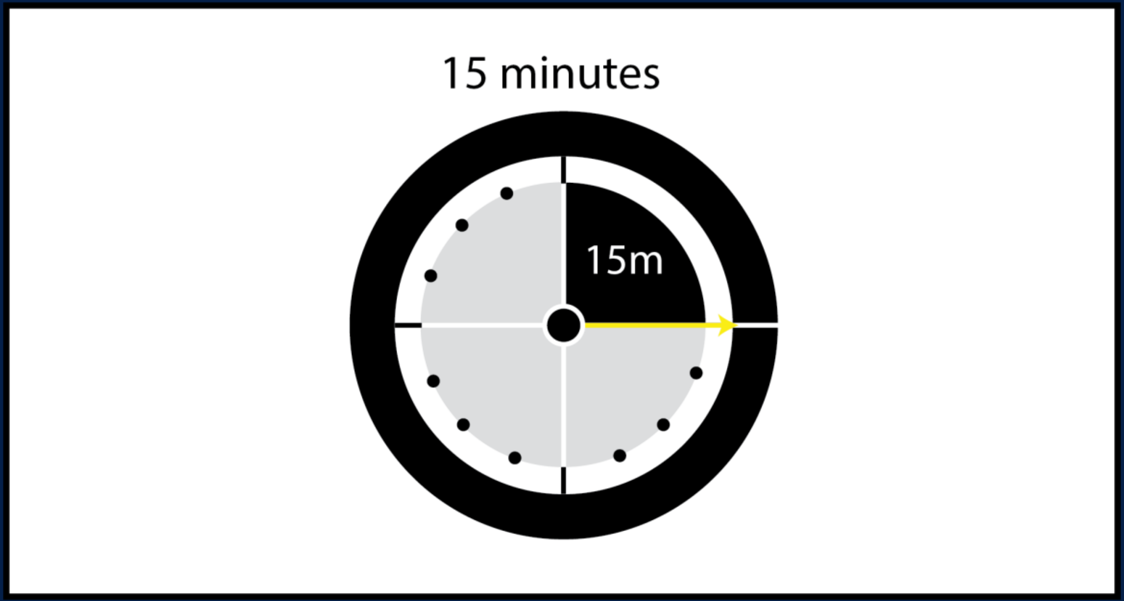

Time to results-2

- The second amount of time is 3 hours. This means that after you provide a sample, the results of your tuberculosis test will be available in 3 hours.

Time to test result-2

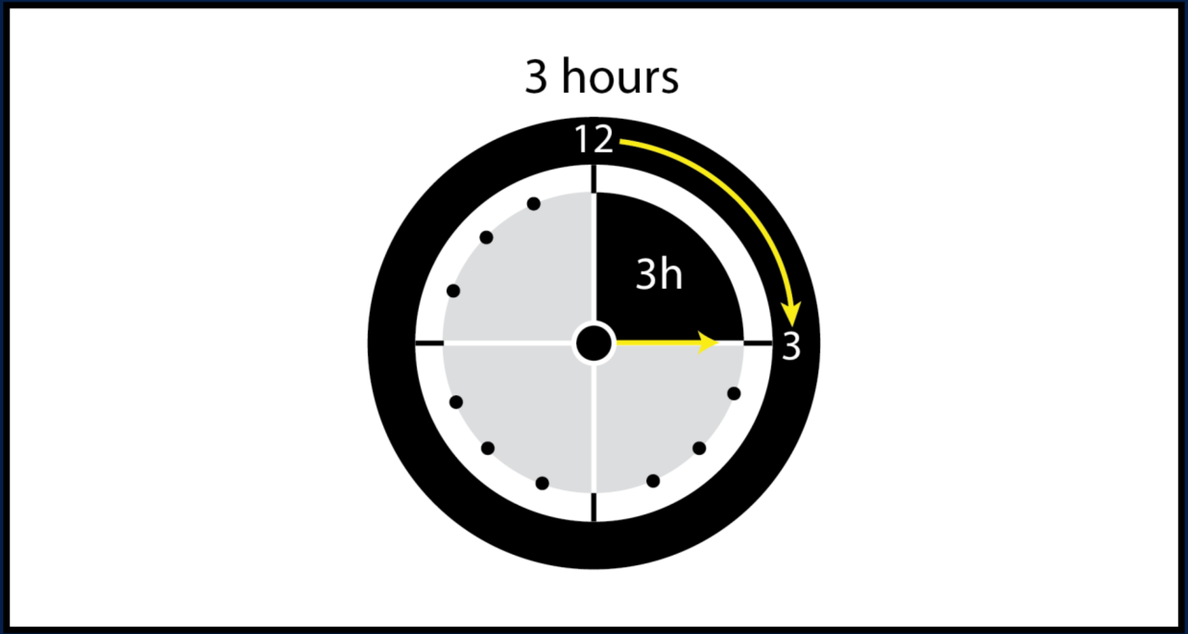

Time to results-3

- The third amount of time is next day. This means that after you provide a sample, the results of your tuberculosis test won’t be available until the next day .

UCSF

Time to test result-3

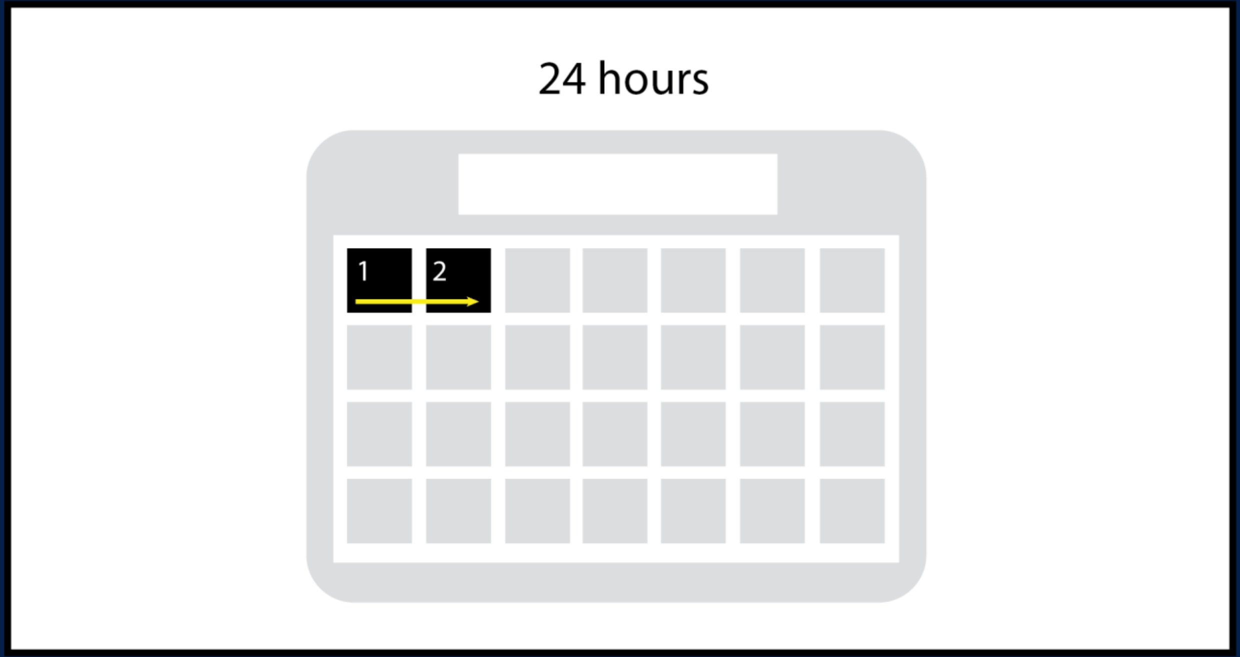

Check understanding

- Do you have any questions about what each picture represents?

Time to test result

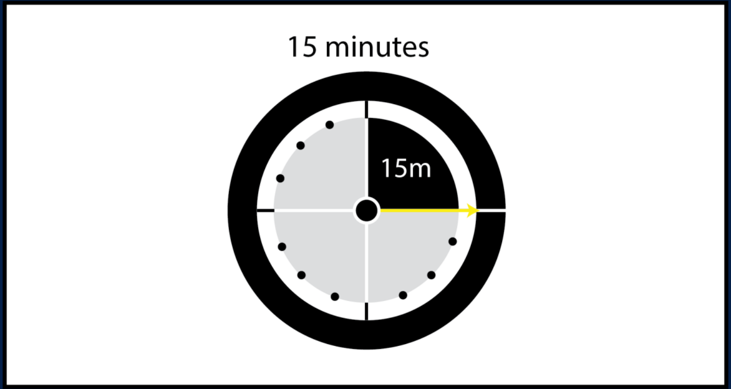

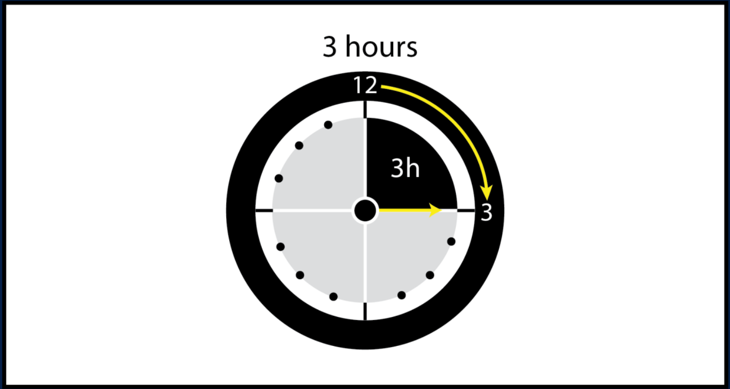

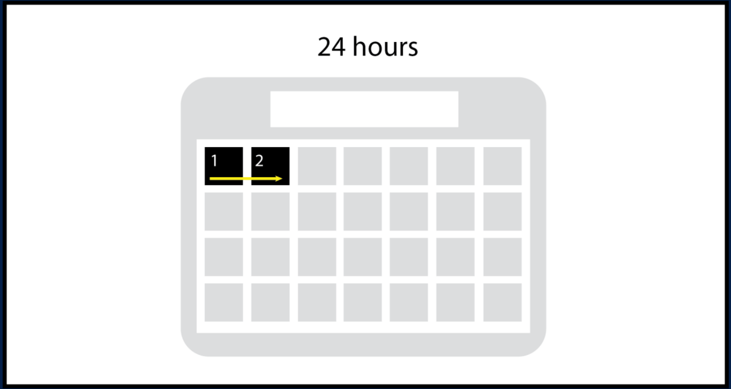

Cost of test

- The fifth option is the cost you need to pay for the test. This is the cost of the test itself that you would need to pay in addition to any costs for travel or time off from work. We will ask you about three different costs that you might pay for a tuberculosis test.

Cost of test

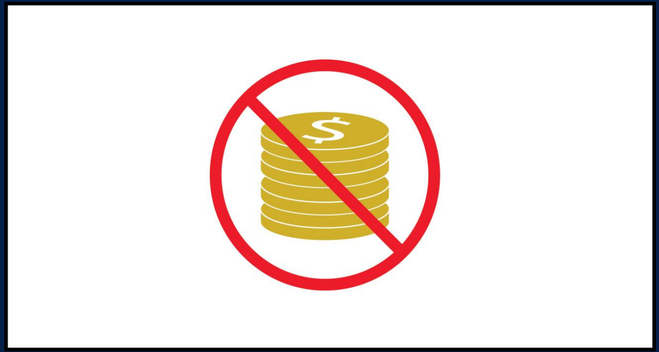

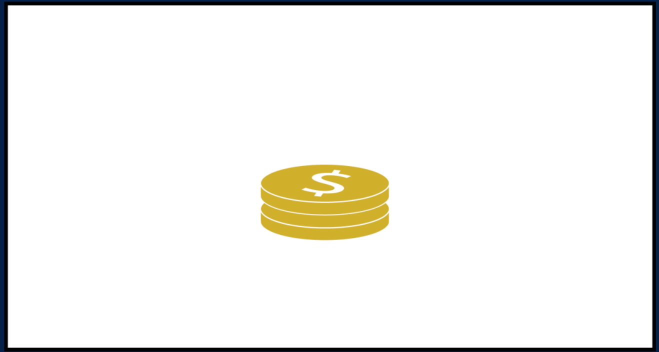

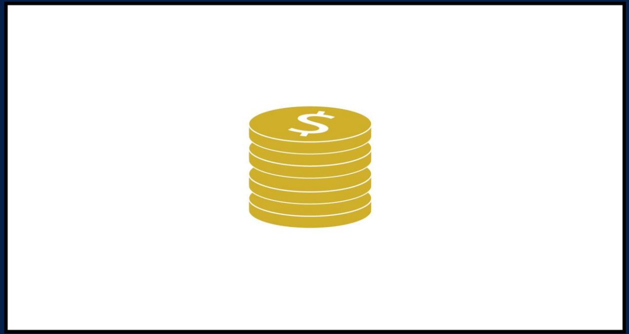

Cost of test-1

- The first amount is **FREE**. This means you would not pay any amount to access a tuberculosis test.

Cost of test-1

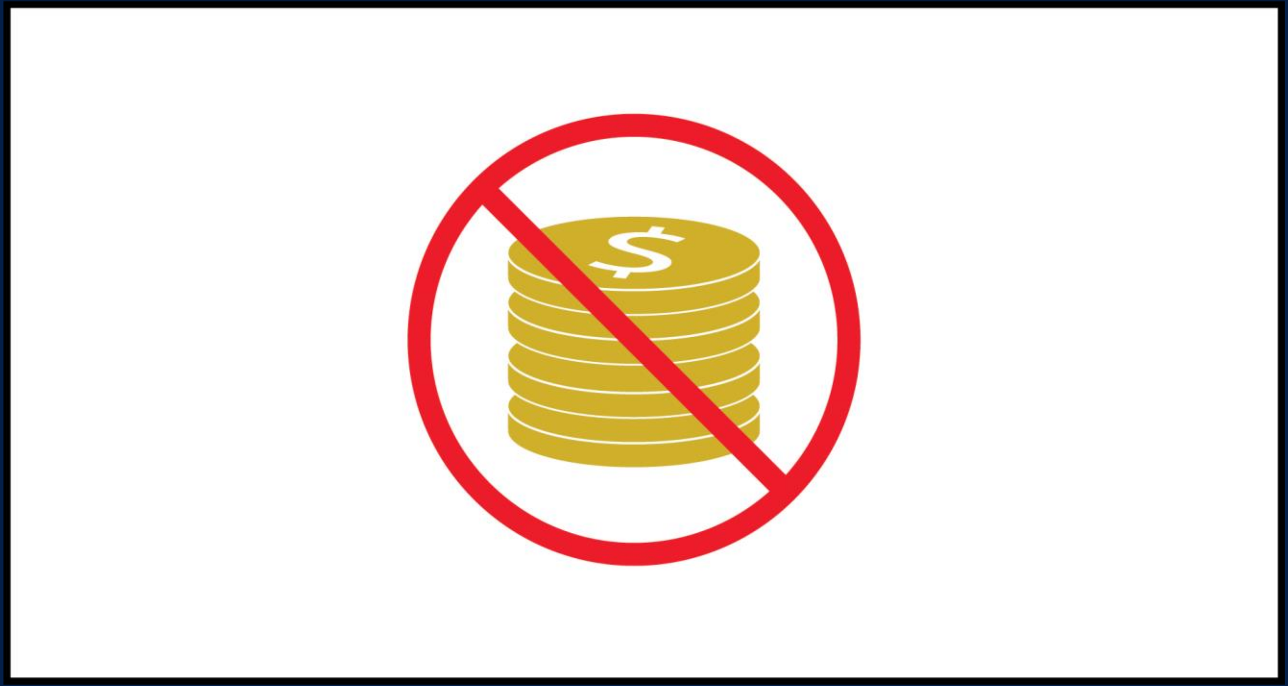

Cost of test-2

- The next amount is **100 pesos**. This means you would pay **100 pesos** to access a tuberculosis test.

UCSF

Cost of test-2

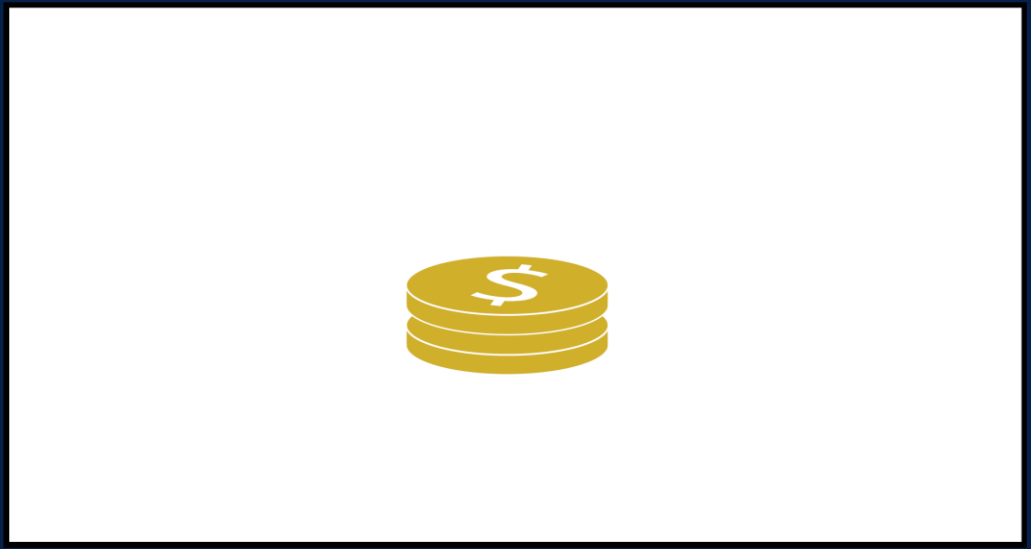

Cost of test-3

- The next amount is **200 pesos**. This means you would pay **200 pesos** to access a tuberculosis test.

Cost of test-3

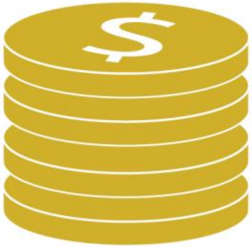

Check understanding of cost

- Do you have any questions about the pictures shown to you?

Cost of test

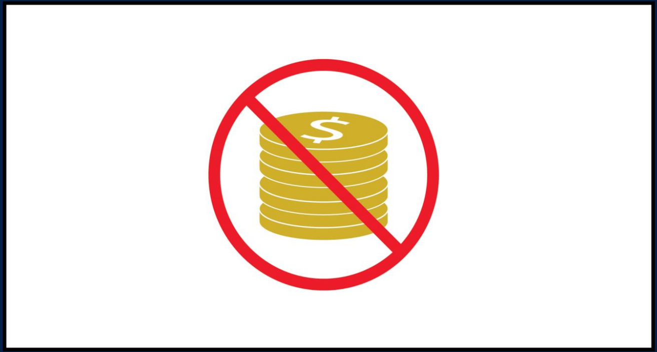

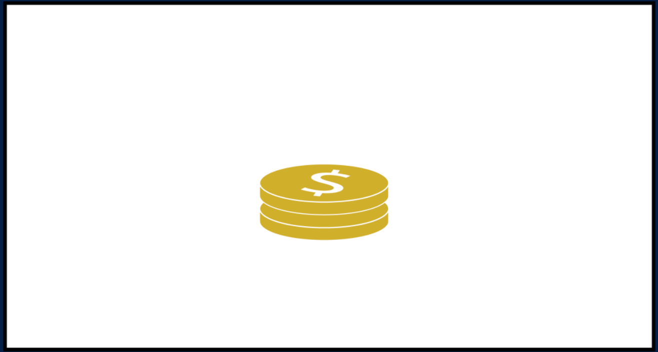

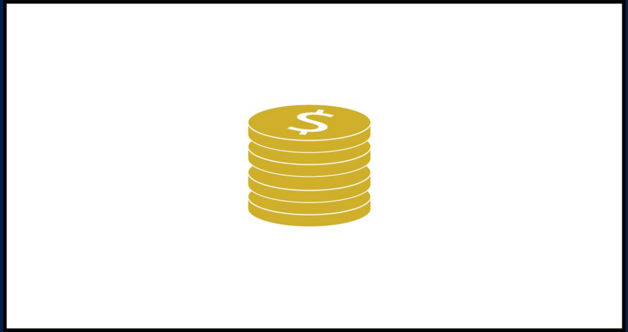

Switch to Sawtooth practice task

- I will now show you an example task of the activity on the study tablet/computer.
